# Unravelling molecular mechanism of oral squamous cell carcinoma and genetic landscape: an insight into disease complexity, available therapies, and future considerations

**DOI:** 10.3389/fimmu.2025.1626243

**Published:** 2025-08-13

**Authors:** Salima Shebbo, Nooralhuda Alateyah, Esraa Yassin, Doaa El Sayed Mahmoud, Faleh Tamimi, Lamyia Anweigi, Abdelbary Elhissi, Haissam Abou-Saleh, Mohamed A. Elrayess, Abdelali Agouni

**Affiliations:** ^1^ College of Dental Medicine, Qatar University (QU) Health, Qatar University, Doha, Qatar; ^2^ Department of Biomedical Sciences, College of Health Sciences, Qatar University (QU) Health, Qatar University, Doha, Qatar; ^3^ Biomedical Research Center, Qatar University (QU) Health, Qatar University, Doha, Qatar; ^4^ College of Medicine, Qatar University (QU) Health, Qatar University, Doha, Qatar; ^5^ Department of Pharmaceutical Sciences, College of Pharmacy, Qatar University (QU) Health, Qatar University, Doha, Qatar

**Keywords:** oral squamous cell carcinoma (OSCC), molecular pathways, genetic predisposition, immunotherapy, therapeutic challenges

## Abstract

Oral squamous cell carcinoma (OSCC) is a heterogeneous malignant neoplasm characterized by intricate molecular pathways and a varied genetic landscape, resulting in a diminished 5-year survival rate. Due to this complexity, many trials of emerging therapies are failing to improve the outcome and survival rate of OSCC, posing a great challenge in the management of this cancer. This review examines the key molecular pathways, genetic susceptibility, and the influence of the microbiome in the advancement of OSCC. Furthermore, it analyses contemporary therapeutic approaches, their limitations, and prospects, especially the incorporation of immunotherapy. The discussion will also encompass the difficulties in turning research findings into successful therapeutic applications and enhancing the patient’s quality of life.

## 1 Introduction

According to the GLOBOCAN estimates, in 2022, Lip and oral cavity cancers ranked as the 15^th^ leading cause of death due to cancer and resulted in 188,230 deaths per year (1). The most common type of cancer in the oral cavity is the squamous cell carcinoma. This malignancy, which accounts for 90% of oral cancers, arises from the epithelial lining of the larynx, pharynx, and oral cavity (2). Oral squamous cell carcinoma (OSCC) is classified under the category of head and neck carcinoma (3).

OSCC causes functional impairments and disfigurations, which include taste, speech, and swallowing difficulties that substantially influence the patient’s quality of life (4, 5). In addition, notable rates of suicidal attempts and ideations have been observed among patients with oral cancers (6).

The development of OSCC is primarily associated with excessive alcohol and tobacco consumption (2), along with other risk factors such as genetic predisposition, epigenetic factors, viruses such as Human papillomavirus (HPV) and Epstein–Barr virus (EBV), gender, race, age, diet, nutrition and consumption of betel nuts, Fanconi anemia and dyskeratosis congenita, as well as immune disorders and certain immunosuppressive agents (7). Furthermore, chronic mucosal trauma due to ill-fitting dentures, sharp teeth, faulty restorations, or implants, and intraoral contact with metal dental restorations were reported to increase the risk of OSCC (8, 9).

OSCC is commonly treated with surgery, chemotherapy, and radiotherapy, depending on the stage. However, over the past few years, immunotherapy (e.g., programmed death-1 (PD-1) immune checkpoint inhibitors) has also emerged as a potential therapy in refractory and relapsed Head and neck squamous cell carcinoma (HNSCC) (10).

Despite the advancements in the treatment modalities of OSCC, there has been no noticeable 5-year survival improvement in recent years. In comparison to other cancers, OSCC was found to have a poor 5-year survival rate of around 35% in India and 50% in Europe. The 5-year-survival rates for this disease overall ranged between 80% for localized, early detected cancers to 20% in the case of regional lymph node involvement, driven by the stage at which the disease was diagnosed (10–12). A recent study reported an approximate 59.3% 5-year survival rate of OSCC, with around 45.7% of cases experiencing metastasis or postoperative recurrences (13). In addition, a recently published, long-term, retrospective observational study that evaluated OSCC risk factors and epidemiology in patients with invasive OSCC over 39 years indicated that 40% of the patients developed recurrence and that the survival rate did not improve over time (14). Furthermore, the Global Cancer Observatory (GCO) has projected that by the year 2040, the incidence of OSCC would increase by roughly 40%, along with a growth in mortality (15). This lack of progress in our management of the disease is attributed to a gap in understanding the complex challenges associated with the genetics, molecular pathways, and treatment modalities of OSCC and their limitations. Therefore, we aim in this review to explore the complex relationship between the genetic landscape and with underlying key molecular pathways of OSCC, their mechanisms, and crosstalk in OSCC. We also provide an overview of positive and detrimental risk factors of the disease, the currently available treatment modalities, and their limitations, and offer insights into the translation of research findings into applicable clinical practices, allowing for tailored personalized medicine.

## 2 Genomic alterations and pathway dysregulation in OSCC

Next-generation sequencing (NGS) offers comprehensive details about the mutations defined in OSCC. These studies investigate the different genetic changes that happen in OSCC and how they affect tumor suppressors, oncogenes, and epigenetic regulators (16, 17). Out of the many OSCC common mutations, the *TP53* (tumor protein 53) gene is one of the most frequently altered. In about 60% to 80% of OSCC cases, alterations occur in *TP53* that lead to oncogenesis (18), resulting in the uncontrolled division of malignant cells, as well as genomic instability, immune suppression, and treatment resistance. These genomic modifications are detected in the early stages of OSCC (19, 20). About 70% of the cases that were examined had genetic alterations in the receptor tyrosine kinases (RTKs), mitogen-activated protein kinase (MAPK), and Phosphoinositide 3-Kinase (PI3-K) signaling pathways (19). RTKs, particularly those from the ERBB family—Epidermal Growth Factor Receptor and ERBB2-4—often drive epithelial cancers. Ligand-binding RTK phosphorylation leads to the activation of various downstream signaling pathways, like the RAS-RAF-MEK-ERK MAPK pathway responsible for cell growth and survival (21). Amplification or somatic mutations were cited as common in RTKs (19) and were common in OSCC samples (21). However, although mutations in RTKs, MAPK, and PI3-K are linked to a poor prognosis, survival outcomes are influenced by multiple factors, including treatment history and immune microenvironment composition, requiring cautious interpretation (21). PI3-K signaling is controlled by oncogenic mutations in *PIK3CA* (Phosphatidylinositol-4,5-Bisphosphate 3-Kinase Catalytic Subunit Alpha) that are often found in OSCC, though the rates of occurrence vary by race (22, 23). These mutations increase the kinase activity, which results in the constitutive phosphorylation of Ak strain transforming (Akt) or Protein Kinase B (PKB), leading to oncogenic transformation (24). Functional research corroborates mutation assertions in Akt activation, establishing it as a therapeutic target, irrespective of the variability in the reported incidence of *PIK3CA* mutations. The alteration in OSCC is also noted to be NOTCH signaling mediation (25, 26), which underlines the further complexity of its role in tumor development (27). Some populations show positive *NOTCH1* variation mutations in a range of 18% to 45% (28, 29). A study of Japanese OSCC patients found that *NOTCH1* mutations were the second most common change, occurring in 25.5% of the time. This is higher than the 17.8% frequency observed in the HNSCC dataset (19). Several studies suggest that alterations in the oncogene *NOTCH1* can act either as tumor-promoting or tumor-suppressing, depending on the cell type. Loss-of-function mutations in *NOTCH1*, *NOTCH2*, *NOTCH3*, and SRY-Box Transcription Factor 2 (*SOX2*) (30) inhibit squamous differentiation, subsequently leading to cancerous growth. Moreover, alterations in *FBXW7*, which regulates NOTCH activity via degradation, further destabilize the Notch system (31). Whole-exome sequencing of OSCC samples has shown frequent alterations in *FAT1* (FAT Atypical Cadherin 1) (32) and *CASP8* (Caspase 8) (33). These mutations have been suggested to increase the likelihood of OSCC proliferation, migration, and resistance to apoptosis (31). Beyond classical oncogenic signaling, alterations in chromatin remodeling genes underscore the role of epigenetic dysregulation in OSCC heterogeneity. Chromosome accessibility and transcriptional regulation are affected by mutations in *KMT2D* (lysine-specific methyltransferase 2D), also known as Mixed-Lineage Leukemia 2 (*MLL2*), a histone methyltransferase that causes H3K4 (Histone 3 Lysine 4) methylation (34). Although their exact role in OSCC is unknown, further mutations in *SYNE1* (Syne Homolog 1) and *TAFIL* [TATA-Box Binding Protein (TBP)-Associated Factor 1-Like] are linked to chromatin remodeling abnormalities (19). Notably, mutations in *MLL*, *MLL3*, and other histone modifiers are frequently detected in HPV-associated oropharyngeal cancers, suggesting a potential virus-driven epigenetic reprogramming mechanism (35, 36). The long-tailed distribution of low-frequency mutations in chromatin regulators reveals the genetic complexity of OSCC, requiring functional validation of epigenetic targets (19). Further mutations in *SYNE1* and *TAF1L* are linked to chromatin remodeling problems (37), but no one knows exactly what role they play in OSCC. Notably, changes in *MLL*, *MLL3*, and other histone modifiers are often found in oropharyngeal cancers that are linked to HPV. This suggests that the virus may change epigenetics (35, 36). The long-tailed distribution of low-frequency mutations in chromatin regulators reveals the genetic complexity of OSCC, requiring functional validation of epigenetic targets (37).

## 3 Epigenetics of OSCC

Epigenetics plays a detrimental role in the development, progression, and treatment resistance of OSCC. It changes the way genes are expressed without interfering with the DNA sequence. These changes encompass DNA methylation, histone modifications, and miRNA regulation (38, 39).

### 3.1 DNA methylation

DNA methylation involves the addition of a methyl group to cytosine residues in CpG islands, habitually steering to gene silencing. In OSCC, aberrant DNA methylation patterns are witnessed in the hypermethylation of tumor suppressor genes (TSGs) and hypomethylation of oncogenes (40).

#### 3.1.1 Hypermethylation of TSGs

Hypermethylation of CpG islands in tumor suppressor gene (TSG) promoters silences their expression, accelerating tumor growth and progression. In OSCC, several genes are frequently hypermethylated (41, 42). Cell cycle regulators, such as *p16* (*CDKN2A*) and *p15* (*CDKN2B*), are key regulators of the G1/S checkpoint, and their silencing leads to unchecked cell cycle progression. Apoptosis-related genes, including *DAPK1* and *p73* (Tumor protein 73), are inactivated through hypermethylation, disrupting apoptotic pathways and promoting tumor survival. DNA repair genes, such as *MGMT* and *MLH1* (MutL homolog 1), play crucial roles in DNA repair, and their silencing contributes to genomic instability, driving carcinogenesis.

#### 3.1.2 Hypomethylation of oncogenes

Conversely, hypomethylation activates oncogenes by increasing their transcription. EGFR (Epidermal Growth Factor Receptor) overexpression, driven by hypomethylation, activates oncogenic pathways such as RAS/RAF/MEK/ERK and PI3-K/Akt/mTOR, which promote tumor proliferation, survival, angiogenesis, and resistance to therapy (43). Survivin, an anti-apoptotic protein, is upregulated through hypomethylation, enhancing tumor aggressiveness by inhibiting programmed cell death (44).

### 3.2 Histone modifications

Histone modifications regulate chromatin structure and gene expression by altering the accessibility of DNA to transcriptional machinery. These modifications, including acetylation, methylation, and phosphorylation, are crucial in OSCC epigenetics (45).

#### 3.2.1 Histone acetylation

Histone acetylation, regulated by histone acetyltransferases (HATs) and histone deacetylases (HDACs), plays an imperative role in gene expression by restraining chromatin structure. HATs add acetyl groups to lysine residues on histones, leading to chromatin relaxation and transcriptional activation, while HDACs remove these groups, triggering chromatin condensation and gene repression (45). In OSCC, the acetylation of histones H3 and H4 is particularly noteworthy, as dysregulated acetylation patterns are concomitant with tumor progression (46, 47). Moreover, HDAC inhibitors (HDACis), such as Trichostatin A (TSA) and Vorinostat (SAHA), have revealed potential in reestablishing normal acetylation levels and resuscitating TSGs, proffering a promising therapeutic approach (45).

#### 3.2.2 Histone methylation

Histone methylation can either activate or repress gene transcription, reliant on the specific lysine residue modified. For example, H3K4me3 is correlated with active transcription, and its dysregulation may endorse oncogene expression, while H3K9me2 is connected to transcriptional repression, with increased levels associating with poor prognosis in OSCC. Additionally, abnormal histone methylation patterns, driven by dysregulated histone methyltransferases (HMTs) and demethylases (HDMs), contribute to OSCC aggressiveness. Furthermore, overexpression of enhancer of zeste homolog 2 (EZH2), a histone methyltransferase, is mostly associated with poor prognosis and increased invasiveness. Targeting these enzymes with inhibitors like DZNep has shown promise in preclinical studies, emphasizing histone methylation as a potential therapeutic target in OSCC (40, 48–50).

#### 3.2.3 Histone phosphorylation

Phosphorylation of histones H3 and H4 plays an imperative role in OSCC progression by influencing chromatin dynamics, mitotic processes, and transcriptional regulation. Histone H3 phosphorylation at serine 10 (H3S10ph) is linked to both chromosome condensation during mitosis and transcriptional activation, with low levels associated with cervical lymph node metastasis. Similarly, histone H4 phosphorylation at serine 1 (H4S1) and high levels of H4K12 acetylation (H4K12ac) are linked with metastasis, gender, and alcohol consumption. These modifications influence chromatin remodeling, gene regulation, and cell cycle control, making them potential biomarkers for OSCC prognosis (46, 51).

### 3.3 MicroRNA dysregulation

miRNAs are small non-coding RNAs that regulate gene expression post-transcriptionally, and their dysregulation plays a significant role in OSCC progression. Oncogenic miRNAs (oncomiRs), such as miR-21 and miR-181b, are overexpressed in OSCC, encouraging malignant transformation by downregulating the mRNA expression of tumor suppressors like *PTEN* (Phosphatase and Tensin Homolog) and *PDCD4* (Programmed Cell Death 4) and heightening inflammation-driven tumor progression. Conversely, tumor-suppressive miRNAs, including miR-133a/b and miR-34a, are often downregulated, escorting oncogene activation, such as PKM2 (Pyruvate Kinase M2)-driven glycolysis and Bcl-2 (B-cell leukemia/lymphoma 2 protein)-mediated apoptosis resistance. In Addition, miRNAs contribute to immune evasion, with miR-200c downregulation facilitating Epithelial-Mesenchymal Transition (EMT) and immune resistance through PD-L1 (Programmed death-ligand 1) modulation. Epigenetic silencing via hypermethylation further exacerbates miRNA dysregulation, highlighting their potential as both biomarkers and therapeutic targets in OSCC (52–55).

## 4 OSCC risk factors and preventive measures

Several risk factors were identified to contribute to the development of OSCC (56). Conversely, there are various protective factors and strategies that can serve as preventive measures against OSCC, and these will be explored in this section (57).

### 4.1 Risk factors

The most relevant among all is tobacco use and alcohol consumption, accounting for 70-80% of all HNSCCs (7). Their combined effect can synergistically increase the risk of oral cancer by around 35% (58). Tobacco smoking inhibits systemic immune functions and causes epigenetic alterations in the oral epithelial cells. In addition, its metabolites can initiate oxidative stress and induce oral squamous cell carcinoma in tissues (59). Tobacco has been shown to activate pathways implicated in OSCC. This includes for instance GLUT-1 (glucose transporter 1), which plays a role in enhancing cancer cell growth and survival, p16 (AKA cyclin-dependent kinase Inhibitor 2A (CDKN2A) (60), which is involved in cell cycle progression and proliferation, and p53 (tumor protein 53), which is important for DNA repair and cell cycle arrest. In addition, PI3-K (phosphatidylinositol 3-kinase) which is involved in cell survival, proliferation, and metabolism, DAPK (death-associated protein kinase), which has a role in tumor suppression and apoptosis, and MGMT (O6-methylguanine-DNA methyltransferase), which is involved in cell genomic stability and DNA repair are also implicated in OSCC (59).

Ethanol contained in alcoholic beverages is classified as carcinogenic (61). OSCC has been inextricably connected with the consumption of alcohol (62). The molecular mechanisms by which alcohol contributes to OSCC include oxidative stress-mediated DNA damage, inflammation, and immune dysregulation through the production of chemokines and cytokines (63), epigenetic modulations (64), and a modification of pivotal signaling pathways implicated in cell proliferation, angiogenesis, and apoptosis, such as the Nuclear Factor-kappa B (NF-κB) and Wnt signaling pathway/beta-catenin (Wnt/β-catenin) (62).

Genetic and epigenetic factors have an important association with OSCC. The primary genes identified earlier to be up-regulated significantly in OSCC early stages are *CPA6* (Carboxypeptidase A6), *TNC* (Tenascin-C), *FMO2* (Flavin-Containing Monooxygenase 2), and *SIAT1* (Sialyltransferase 1). In addition, the expression of the *LGI1* (Leucine-Rich, Glioma-Inactivated 1) gene was found to be enhanced in healthy surrounding mucosa, nevertheless, it did not show differential expression in cancerous tissue biopsies (65). More recent studies indicated other genetic contributors to the development of HNSCC, including several DNA repair enzymes, Fanconi anemia-associated genes, apoptotic pathway members, and cell-cycle control proteins, which were suggested to modulate HNSCC susceptibility (66).

It is crucial to emphasize the risks associated with using heavy metals such as titanium, chromium, gold (67), silver, cobalt, palladium, nickel, tin, and copper, as well as alloys containing silver-tin and mercury, which are commonly used in dental amalgams (68). For instance, chromium and cadmium are reported to induce oxidative deterioration in biological macromolecules, and cadmium can replace zinc in the DNA binding domains of zinc finger and hence trigger cancer (69, 70). Senevirathna et al. observed significantly higher cadmium, chromium, copper, cobalt, and zinc serum concentrations in patients with OSCC and oral potentially malignant disorders (OPMD) (69). A recent *in vitro* study demonstrated that iron and nickel induce the generation of reactive oxygen species (ROS), whereas manganese and chromium were associated with both ROS production and single-strand breaks in DNA. The cytotoxic effects identified in the analyzed eluates were attributed to iron, aluminum, manganese, and chromium (71). The accumulation of heavy metals in saliva can lead to DNA damage, cytokine production, oxidative stress, and inflammation. Silver, chromium, and nickel have been shown to suppress fibroblast proliferation. Elevated levels of metal ions such as nickel, cobalt, titanium, and chromium are associated with hypersensitivity reactions, chronic inflammation, cell death, and mutations. Notably, some studies have reported that Ni^2+^ induces the production of inflammatory genes, such as interleukin (IL)-8, in primary human monocytes (72). Other major risk factors of OSCC include genetic predisposition, viruses such as HPV and EBV, gender, race, age, diet, nutrition, and consumption of betel (Arecal) nut (7). According to the GCO, OSCC appears to affect males more than females, and the susceptibility increases in men ranging from middle-aged to elderly (3). However, recent reports indicate a rising trend in OSCC cases among patients under the age of 45 (73). Furthermore, the use of Areca nut was found to have a significant correlation with OSCC. This phenomenon was predominantly observed in the Pacific and East Asian populations (15). Furthermore, current data support the association of Fanconi anemia and dyskeratosis congenita, wherein OSCC is predominantly inherited. A correlation between immune disorders, such as human immunodeficiency virus (HIV) in smokers and immunosuppressive medications, and HNSCC has been demonstrated, as both are considered possible risk factors (7).

### 4.2 Protective factors and preventive measures

**Figure 1:**
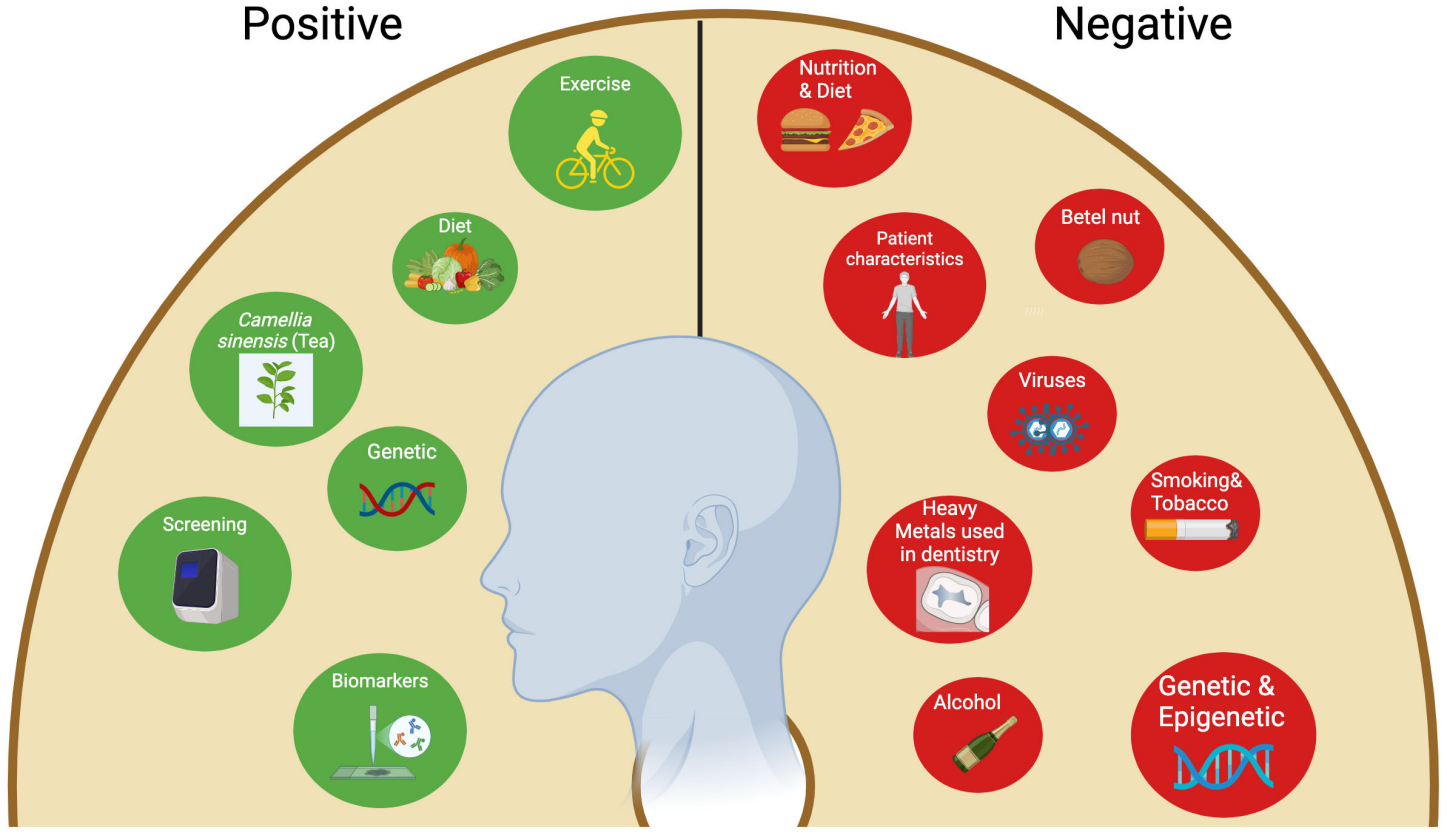
Negative and positive (Protective) factors associated with oral squamous cell carcinoma.

Conversely, several preventive strategies to address this hazardous situation have been documented. A portion of them are factors susceptible to change through lifestyle improvements. The others are impervious and genetic. The former mostly entails avoiding modifiable deleterious risk factors such as tobacco use, alcohol intake, and betel quid (57). Furthermore, maintaining a balanced, healthy diet that contains fresh vegetables and fruits, good mouth hygiene practices, avoiding direct exposure to the sun, and regular exercise are all advisable practices (74, 75). The mechanism by which dietary regimens mitigate the risk of oral cancers is believed to be by affecting metabolic genes and detoxifying enzymes (74). Some studies reported that tea (*Camellia sinensis*) comprises components able to inhibit oral cavity carcinogenesis via a mechanism involving the reduction of cell proliferation and induction of cell apoptosis (76, 77). Another pivotal preventive measure for oral cancers is performing screening tests such as VEL scope, Orascoptic DK, Micolux/DL, VizilitePlus, Vizilite, and OralCDx. These screening tools assist in the prediction and early detection of oral cancers (78). The utilization of efficient biomarkers is a crucial element in the early detection of oral malignancies. These encompass processes, substances, or structures quantified in products or the body that can forecast or affect the occurrence of an event. Biomarkers associated with oral cancer encompass salivary indicators for OSCC diagnosis, including sphinganine, S-carboxy-methyl-L-cysteine, phytosphingosine, and L-phenylalanine. Furthermore, at the genetic level, *CD34* expression serves as a marker for recurrence detection, whereas the genomic markers *ITGB4* (Integrin Subunit Beta 4) and *ITGA3* (Integrin Subunit Alpha 3) are regarded as drivers of distant metastasis and prognosis (57). In Europe, a multicenter study conducted by Hashibe et al. revealed a positive relationship between the allele ADH1B*2 and alcohol drinkers, as it highlighted a protective effect of this allele in drinkers in comparison to the allele ADH1B*1/*1 (79). **Figure 1** summarizes the risk factors associated with OSCC.

## 5 Molecular mechanisms underlying OSCC

**Figure 2:**
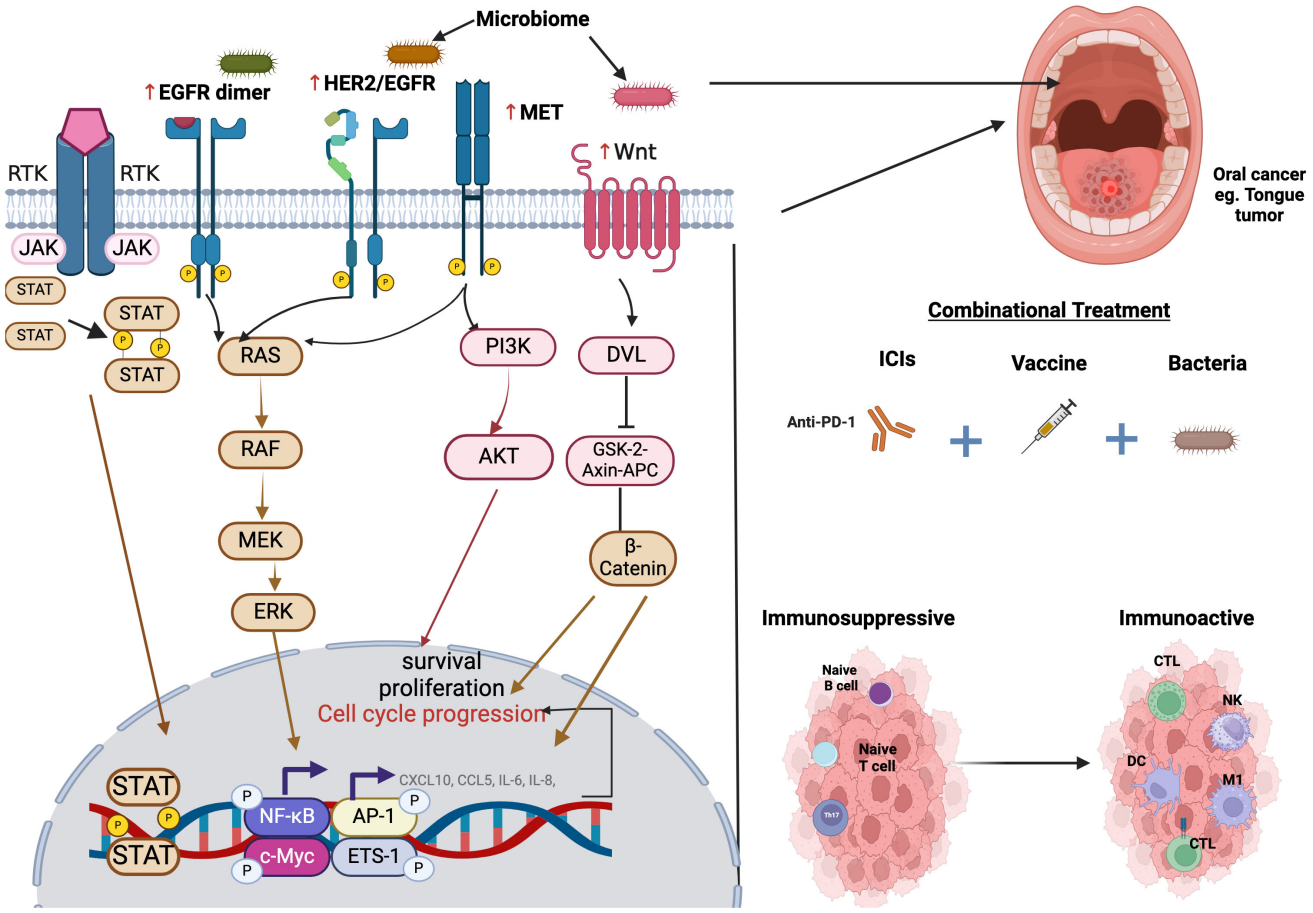
Illustration of the molecular pathways implicated in cancer initiation and progression. The microbiome and other signals influence receptor tyrosine kinase (RTK) signaling, leading to the activation of EGFR, HER2/EGFR, and MET, which in turn trigger downstream pathways such as JAK-STAT, RAS-RAF-MEK-ERK, PI3-K-Akt, and Wnt/β-catenin signaling. These pathways promote cell survival, proliferation, and cell cycle progression, contributing to tumorigenesis. Additionally, a combinational treatment approach incorporating beneficial bacteria, immune checkpoint inhibitors (such as anti-PD-1), and cancer vaccines may help transform an immunosuppressive or “cold” tumor into an immune-active or “hot” tumor. This transformation is characterized by enhanced infiltration of cytotoxic T lymphocytes (CTLs), natural killer (NK) cells, dendritic cells (DCs), and M1 macrophages, ultimately fostering a more effective anti-tumor immune response.

OSCC can occur through many molecular mechanisms, such as genetic mutations in oncogenes and tumor suppressor genes, which will lead to uncontrolled cell proliferation and evasion of apoptosis, epigenetic modifications, which play an important role in the regulation of gene expression in OSCC, thus lead to the silencing of tumor suppressor genes and activation of oncogenes. Chronic Inflammation also contributes to the development and progression of OSCC. Inflammatory cytokines and growth factors promote tumor growth and angiogenesis. Epithelial-mesenchymal transition is aided by various signaling pathways and transcription factors, considered a key step in cancer metastasis. Angiogenesis, as OSCC cells secrete angiogenic factors that stimulate the growth of blood vessels, provides the tumor with nutrients and oxygen. Immune Evasion as OSCC cells can evade the immune system through various mechanisms, which allows the tumor to escape immune surveillance and continue to grow unnoticed. Metabolic Reprogramming, where Cancer cells often undergo metabolic changes to support their rapid proliferation, can be targeted for therapeutic interventions. Microenvironment interactions, as crosstalk between cancer cells and the microenvironment, can promote tumor growth, invasion, and metastasis. Understanding these complex molecular mechanisms is essential for developing targeted therapies and improving the prognosis for patients with OSCC (80–84). **Figure 2** summarizes the main molecular pathways implicated in the initiation and progression of OSCC.

### 5.1 G-Protein coupled receptor-related pathways: Wnt/B-catenin-EGFR pathway

#### 5.1.1 Wingless/integrated and beta-catenin signaling pathway

The Wnt/β-catenin pathway is a critical signaling pathway that plays a role in various physiological processes, including embryogenesis and tissue regeneration. This pathway is tightly regulated in the human body, as its dysregulation leads to cancer (85). The Wnt pathway diverges into canonical (β-catenin dependent) and non-canonical (β-catenin independent) (86). In the absence of the Wnt signal, the scaffolding protein disheveled (DVL) is not recruited to the Frizzled receptor. This allows the destruction complex [Axin (Axis inhibition protein), GSK-3 (Glycogen synthase kinase 3), and APC (Adenomatous Polyposis Coli)] to sequester β-catenin in the cytoplasm and target it for degradation (86). Conversely, in the presence of the Wnt signal, disheveled is activated by Frizzled receptor (FZD) and co-receptor LRP5/6. Consequently, the destruction complex is dissociated, and β-catenin will be translocated to the nucleus, interacting with TCF/LEF to open genes involved in cell cycle progression and proliferation (86). Many studies highlighted the role of the Wnt pathway in OSCC progression, invasion, and migration, which is mediated through aberrant regulation of β-catenin (87–89). Wnt1 signaling activates the canonical pathway, inhibits apoptosis, and promotes cell invasion in OSCC (90). Li et al. demonstrated that OSCC progression, invasion, and migration are potentially initiated by Wnt7B (non-canonical) through Wnt/β-catenin activation, and its abrogation reverses these effects (91). Another Wnt subtype, Wnt 7A, is stimulated by EGF through *Akt*, promoting the Wnt7A/β-catenin pathway to enhance Matrix metalloproteinase-9 (MMP9) expression. These events stimulate cell invasion in OSCC (92). In HNSCC, Huang et al. suggested that Wnt7A is associated with cancer progression and survival independently of β-catenin, instead activating FZD7/JAK1 (Janus Kinase 1)/STAT3 (Signal Transducer and Activator of Transcription 3) axis (93). Collectively, these studies provide evidence of potential prognostic markers and a therapeutic target for precision medicine; however, further studies are needed to elucidate the molecular pathways through which Wnt7A and Wnt7B exert their effects. A study investigating the role of *CDK5RAP2* (Cyclin-Dependent Kinase 5 Regulatory Subunit Associated Protein 2) in OSCC demonstrated that Wnt/β-catenin stimulates OSCC progression and cell pluripotency through CDK5RAP2 (94). This study highlights a stemness-associated prognostic factor and provides insight into the regulation of pluripotent cell populations in OSCC. Independent of Wnt/β-catenin activation and E-cadherin downregulation, Wnt5A, a non-canonical activator, enhanced OSCC progression. In this study, Prgomet et al. identified Wnt5A as a potential biomarker for the progression of OSCC from dysplasia to oral cancer (95). A pioneering study examined the link between β-catenin expression and immune cell infiltration in OSCC and showed that accumulated nuclear β-catenin is associated with a reduction in CD8+ Tumor-infiltrating lymphocytes (TIL) in the tumor microenvironment (TME), particularly in the stroma and tumor (96). Furthermore, β-catenin expression correlated with low PD-L1 tumor expression, indicating immune evasion and fostering an immunosuppressive TME (96). The results of this study warrant further investigation, underscoring a pivotal pathway through which cancer can be targeted and eradicated. Mounting evidence emphasizes the interplay between Wnt/β-catenin, the microbiome, and cancer (97–99). In OSCC, an *in vivo* study demonstrated that *Fusobacterium nucleatum* (*F. nucleatum*), through the CDH1/β-catenin pathway, enhanced OSCC proliferation (100). Another study revealed a sophisticated interplay between host oral microbiota and imbalanced gene expression in OSCC development. This multi-omics study identified 20 perturbed CpG methylated promoter regions in host genes that were associated with an increased abundance of harmful bacteria in tumors. Furthermore, *in vitro* work in the same study uncovered the mechanism by which *F. nucleatum* induced tumor dissemination. This occurred through upregulating the *SNAI2* (Snail Family Transcriptional Repressor 2) gene, which resulted in crosstalk between the E-cadherin/β-catenin pathway, tumor necrosis factor (TNF)-α/NF-кB signaling, and extracellular remodeling, leading to EMT (101). Zhou et al. demonstrated that intra-tumoral colonization with pathogenic bacteria, such as *Streptococcus mutans*, enhances the production of onco-metabolites like kynurenic acid and IL-1β that lead to T cell exhaustion in TME and undermine the effectiveness of immune checkpoint blockade (anti-PD-L1) and IL-1β inhibitors (102). Consequently, modulating the oral microbiota, targeting its metabolites, and unraveling the interplay between the oral microbiome, the TME, host gene expression regulation, and underlying molecular pathways could serve as a prophylactic and therapeutic intervention for OSCC.

#### 5.1.2 The EGFR pathway

The transmembrane EGFR receptor belongs to the ErbB family of RTKs, which also includes ErbB2 (HER-2/Neu), ErbB3 (HER-3), and ErbB4 (HER-4). EGFR is overexpressed in up to 90% of OSCC cases and plays a crucial role in other malignancies. The activation of EGFR is frequently observed in oncogenic pathways linked to the etiology of oral cancers, worsening survival rates, and elevating the risk of lymph node metastases. Activated EGFR initiates several signaling pathways, including the ERK and STAT3 pathways, which promote cell proliferation, tumor progression, and survival. Dysregulation of the EGFR signaling cascade has been shown to critically affect the progression and development of HNSCCs, including OSCC. Many studies highlight EGFR’s role in OSCC progression and chemoresistance, further emphasizing its oncogenic potential. Any disruption in EGFR is associated with the inhibition of cell proliferation and suppression of oncogenic signaling pathways such as PI3-K/Akt and Myc (Myelocytomatosis Oncogene) in OSCC cells (103–105). Additionally, radiation therapy resistance in OSCC is closely related to *EGFR*-mediated signaling pathways. Radiation can induce EGFR translocation to the nucleus, where it functions as a transcription factor, leading to radio-resistance (106). Furthermore, genetic polymorphism in the EGFR gene, such as rs2227983, predicts increased susceptibility and progression of OSCC (107). Understanding the significance of the EGFR-mediated signaling pathways in OSCC will help in devising more effective treatment methods.

#### 5.1.3 Mesenchymal-epithelial transition pathway

The stimulation of the MET signaling response facilitates tumor development, angiogenesis, and metastasis in numerous cancers. Recent studies indicated that either MET, its ligand *HGF* (hepatocyte growth factor), or both were expressed in 80% of HNSCC cases. Seiwert et al. identified MET overexpression in 84% of the analyzed cases of HNSCC in their study. In contrast, the suppression of MET signaling leads to a decrease in cellular proliferation, migration, motility, and angiogenesis, underscoring its vital role in tumor growth (108).

#### 5.1.4 MET and EGFR crosstalk

The MET signaling pathway interacts with other essential signaling pathways. EGFR and MET function as upstream regulators of the MAPK (RAS/RAF/MEK/ERK) and PI3-K/Akt/mTOR signaling pathways. Multiple systems have documented functional interactions between MET and other receptors, including EGFR, ERBB2, and IGF1R (Insulin-Like Growth Factor 1 Receptor). This interaction is regarded as a pivotal mechanism that initiates cancer growth and confers resistance to therapy. A previous study by Stabile et al. that integrated the EGFR inhibitor erlotinib with the MET inhibitor PF04217903 in a c-SRC (Cellular Sarcoma Kinase) expressing cell line demonstrated a significant reduction in cell viability and tumor volume. The findings indicate that resistance to anti-EGFR therapy, including monoclonal antibodies, may arise from the functional interaction between EGFR and MET in the cells of HNSCC patients. Consequently, there is a growing interest in formulating novel techniques that facilitate the concurrent use of MET and EGFR inhibitors, particularly in instances when MET serves as a targetable co-driver, such as in EGFR-mutant cancers. The implementation of this combinatorial approach may improve therapeutic efficacy and diminish resistance mechanisms (109).

#### 5.1.5 RAS/RAF/MAPK pathway

The RAS/RAF/MAPK pathway is pivotal in the pathophysiology of OSCC since it is active in over 50% of human oral cancer cases, indicating its substantial influence on the pathogenesis and progression of OSCC (110). The RAS/RAF/MAPK pathway is initiated by the binding of growth factors, such as EGF (epidermal growth factor), to RTKs, leading to the activation of RAS proteins. This activation subsequently recruits and phosphorylates RAF proteins, which activate MEK (Mitogen-Activated Protein Kinase Kinase) and ERK (Extracellular Signal-Regulated Kinase), ultimately regulating various cellular processes, including survival, proliferation, and migration (111). The RAS/RAF/MAPK pathway interacts intricately with other signaling cascades in OSCC. The PI3-K/Akt/mTORC1 (mechanistic Target of Rapamycin Complex 1) pathway and the RAS/RAF/MAPK pathway interact, resulting in the dysregulation seen in numerous human malignancies (112). This interaction is mediated by different feedback loops and shared components, such as the GAB docking proteins, which contribute to both RAS activation and PI3-K signaling (21). BRAF protein, a member of the RAF family, plays a significant role in OSCC progression. Unlike *RAS* mutations, *BRAF* mutations specifically initiate the MAPK signaling pathway, which in turn will promote tumor progression (113, 114). This specificity makes *BRAF* a strategic target for OSCC therapies. Recent studies have uncovered complex regulatory mechanisms within the RAS/RAF/MAPK pathway in OSCC. For example, during the process of activation, RAF proteins can form dimers or higher-order multimers, which are crucial for signal transduction and can be targeted for OSCC treatment. Furthermore, the RAS/RAF/MAPK pathway interacts with the PI3-K/Akt/mTOR (Mammalian Target of Rapamycin) pathway, facilitated by several feedback loops and common elements, such as the GAB docking proteins, which play a role in both RAS activation and PI3-K signaling. The complex interplay of positive feedforward and negative feedback loops profoundly influences signal patterns in OSCC cells (112). Understanding the intricate mechanics of the RAS/PAF/MAPK pathway in OSCC is crucial for creating successful targeted therapeutics. Presently, extensive research is concentrated on elucidating the relationships among diverse signaling cascades and integrating therapeutic methods that target multiple pathway components to enhance therapeutic efficacy and circumvent potential resistance mechanisms in OSCC patients.

### 5.2 Inflammatory pathways associated with OSCC

It is predicted that about 15% of all cancers are ascribed to various inflammatory processes, and mounting evidence supports the association between OSCC and chronic inflammation (115). This section will examine the most deleterious inflammatory pathways implicated in the stages of OSCC. This will significantly aid in customizing a targeted therapy that can combat the disease with minimal compromises.

#### 5.2.1 Nuclear factor kappa-light-chain-enhancer of activated B-cells

NF-κB is one of the chief inflammatory transcription factors, and its aberrant expression has been connected to oncogenesis (116). It is extensively expressed in various cancers, and it regulates the expression of a variety of genes in the pathways related to cell survival, inflammation, tumorigenesis, proliferation, and therapeutic resistance (117–120). NF-κB initiates its function upon activation by lipopolysaccharide (LPS), TNF-α, or IL-1 (118). Following activation, the transcription factor facilitates the expression of many cytokines, including IL-8, IL-6, and IL-1, hence predisposing the individual to inflammation. In OSCC, these transcription factors are constitutively activated, resulting in the overexpression of inflammatory genes *CXCL10*, *CCL5*, *IL-6*, and *IL-8*, which are the primary factors responsible for the inflammatory infiltrate observed in the TME (120). NF-κB has a crucial role in defining the malignant phenotype of oral cancer by promoting angiogenesis (121), invasion (122), and metastasis (123).

#### 5.2.2 Activator protein 1

AP-1 is another important transcription factor complex involved in the regulation of cell survival and death. It consists of Jun protein homodimers, or Jun and Fos proteins heterodimers (124), and is activated by the cytokine IL-1. AP-1 regulates the expression of several genes involved in activities such as proliferation, DNA synthesis, cellular invasion, and apoptosis (115, 125, 126). The high expression and activation of this pathway were associated with drug resistance, complicated by the drug therapy regimens (125), making it an interesting target for cancer therapy (126). Upon activation by IL-1, AP-1 triggers the release of IL-8, hence facilitating cell proliferation and survival in HSNCC. Another study indicated that AP-1 may facilitate the activation of *Bcl-2* expression. This is implicated in the resistance to chemoradiation and the inhibition of apoptosis in recurring oral tumors exhibiting radio- and chemo-resistance (127). This indicates that targeting IL-1, which promotes the AP-1 pathway, may overcome the chemoradiation resistance observed in OSCC.

#### 5.2.3 Cytokine receptors JAK/STAT

The Janus kinase/signal transducer and activator of transcription (JAK/STAT) pathway is a key signaling mechanism cytokine receptors use to transmit signals from the cell surface to the nucleus (128). This pathway is critical for cellular growth, differentiation, and apoptosis (129). Aberrant activation of this pathway, particularly STAT3, has been implicated in different oncogenic processes, including cell proliferation, survival, angiogenesis, invasion, metastasis, and immune evasion (130). Concerning cell proliferation and survival in OSCC, the binding of IL-6 to its receptor activates the JAK2/STAT3 signaling pathway, and elevated levels of IL-6 in OSCC tissues are positively associated with the expression of SRY-box transcription factor 4 (Sox4), both of which contribute to OSCC proliferation and survival (131). In OSCC, hyperactivation of STAT3 leads to increased expression of pro-survival and proliferative genes such as *cyclin D1* and *Bcl-xL*. Cyclin D1 promotes cell cycle progression (132), while Bcl-xL is anti-apoptotic; its overexpression raises the threshold for apoptosis, allowing tumor cells to resist various physiological death signals (133). Moreover, Cyclin D1, in conjunction with cyclin-dependent kinases (CDKs) such as CDK4 and CDK6, endorses cell cycle progression by phosphorylating the retinoblastoma protein (pRb), so attenuating transcriptional repression of E2F target genes necessary for DNA synthesis (134, 135). Furthermore, STAT3 wields its pro-proliferative effects by suppressing the transcription of CDK inhibitors such as p21 and p27, which are essential negative regulators of cell cycle progression (136). The downregulation of these inhibitors confiscates critical cell cycle checkpoints, compounding cell proliferation and the likelihood of mutation accumulation (137). For angiogenesis, upon activation, STAT3 binds to the VEGF (Vascular endothelial growth factor) promoter, the most potent pro-angiogenic factor, which results in VEGF production, which results in increased microvessel density (MVD) within tumors by stimulating endothelial cell proliferation, migration, and new blood vessel formation (138). Elevated VEGF levels show a relationship with aggressive OSCC phenotypes and poor prognosis due to enhanced tumor vascularization and metastasis (139, 140). Additionally, the STAT3-VEGF axis was tested by pre-clinical studies, indicating that inhibition of STAT3 downregulates VEGF expression and suppresses angiogenesis in xenograft models (141–143).

The stimulation of the JAK/STAT3 pathway enhances the invasiveness and metastatic potential of tumor cells by: **a)** The activation of STAT3 in OSCC cells results in elevated expression of MMP-9 (matrix metalloproteinase-9), a crucial enzyme that destroys the extracellular matrix (ECM), hence promoting cancer cell invasion and metastasis. IL-22 and IL-6 facilitate OSCC cell migration and invasion through the activation of the JAK/STAT3/MMP-9 signaling pathway (137, 144); and **b)** EMT is a pivotal biological process facilitating OSCC metastasis. STAT3 facilitates EMT in OSCC cells by downregulating E-cadherin, wherein the active JAK2/STAT3 signaling pathway diminishes E-cadherin expression, hence decreasing cell-cell adhesion and fostering a more migratory phenotype. The activation of STAT3 boosts the expression of mesenchymal markers, including N-cadherin and E-box binding zinc finger protein 2 (ZEB2). This alteration in cadherin expression is a characteristic of EMT and increases cell motility (137, 145).

Upon activation, STAT3 signaling modulates immune responses by several mechanisms, which are: **a)** Modulation of myeloid-derived suppressor cells (MDSCs). MDSCs are immunosuppressive cells that hinder T-cell activation and foster tumor growth; the hyperactivation of STAT3 heightens the recruitment and expansion of MDSCs within the TME. What’s more, STAT3 upregulates chemokines like CXCL1 (C-X-C Motif Chemokine Ligand 1) and attracts MDSCs to tumor sites. Hence, inhibition of STAT3 has been shown to reduce MDSC populations, thereby enhancing antitumor immunity (137, 142, 146); **b)** Polarization of tumor-associated macrophages (TAMs). TAMs play a decisive role in cancer progression, they can be polarized into M1 (anti-tumor) or M2 (pro-tumor) phenotypes, with M2 TAMs dominating in the TME (147, 148). STAT3 activation enhances the expression of CD39 and CD73 on monocytes, driving the conversion of extracellular ATP into adenosine, and this inaugurates an immunosuppressive microenvironment that endorses the differentiation of monocytes into MDSCs through the CD39/CD73-adenosine signaling pathway (149). In OSCC tissues, phosphorylated STAT3 (p-STAT3) is positively concurrent with markers of MDSCs, such as CD33 and CD14; high p-STAT3 levels are associated with increased MDSC accumulation and enhanced immunosuppressive activity in the TME (146); **c)** Suppression of Dendritic Cell (DC) Function: STAT3 hyperactivation disrupts DC maturation, weakening their ability to present antigens and activate T cells, while immature DCs accumulate in the TME due to STAT3 signaling and immunosuppressive factors, including regulatory T-cells, tumor-associated macrophages, and hypoxic conditions. These factors cooperatively preserve DCs in an immature state, further aggravating immune suppression in TME (146, 150); and **d)** Downregulation of Cytotoxic T-cell Responses: STAT3 activation plays a fundamental role in suppressing cytotoxic CD8+ T-cell responses, empowering tumor immune evasion, this ensues through the induction of immunosuppressive cytokines such as IL-10 and TGF-β, which create an inhibitory TME that impairs T-cell activity (151). As mentioned earlier, STAT3 also promotes the expression of survival genes like Bcl-xL and cyclin D1, further protecting OSCC cells from immune-mediated destruction (132). Additionally, STAT3 signaling fosters the recruitment of immunosuppressive cells, such as regulatory T-cells and myeloid-derived suppressor cells, while reducing immune-activating cytokines like IL-2 (151, 152). **e)** Upregulation of Immune Checkpoints: Immune checkpoint molecule expression is significantly upregulated by hyperactivation of STAT3, particularly PD-L1, which suppresses T-cell activity and facilitates immune evasion. In OSCC, increased PD-L1 expression is closely linked to heightened STAT3 signaling, as STAT3 directly controls PD-L1 transcription. This interaction creates an immunosuppressive TME by suppressing cytotoxic T-cell activity and facilitating tumor progression (153, 154). Collectively, these mechanisms contribute to OSCC progression and resistance to immune surveillance, highlighting STAT3 as a key therapeutic target for restoring antitumor immunity in OSCC patients.

## 6 Implications of the microbiota in OSCC

OSCC has a distinct microbial signature, while the oral microbiome plays a significant role in its onset. The gut microbiome, although potentially important, is not well-characterized (155). Recent studies revealed reduced microbial diversity and changes in the relative abundance of certain bacterial taxa in OSCC tissues compared to healthy ones. This dysbiosis in the oral environment may contribute to the etiopathogenesis of OSCC (156). The involvement of oral microbiota in OSCC pathogenesis is driven by interconnected mechanisms, including chronic inflammation, direct cellular effects, metabolite production, and modulation of key signaling pathways.

### 6.1 Chronic inflammation

Chronic inflammation is a recognized pathway contributing to OSCC drive (157). It is initiated by the persistence of microbial dysbiosis, which activates the recruitment of pro-inflammatory cytokines and immune cells (158). The relationship between chronic inflammation and dysbiosis appears to be bidirectional: persistent infections that manifest as chronic inflammation can perturb the oral microbiota, leading to dysbiosis. Conversely, dysbiosis, characterized by a shift in the bacterial population toward more pro-inflammatory species, can itself trigger inflammation (159). For example, TNF-α, IL-1, IL-6, and IL-8 have shown potential as diagnostic and prognostic biomarkers for OSCC (115). Among the bacteria linked with inflammation are *Porphyromonas gingivalis* (*P. gingivalis*) and *F. nucleatum*, both of which are known to induce the production of pro-inflammatory cytokines such as TNF-α, IL-1, IL-6, IL-8, as well as activation of NF-κB and NLRP3 pathways (160–162). Therefore, mitigating and controlling inflammation, as well as stabilizing the microbiome landscape, is key to reducing the risk of OSCC initiation from these causes.

### 6.2 Direct cellular effects

A major direct cellular effect of the oral microbiome in OSCC is the stimulation of cell proliferation and invasion. Hebshi et al. identified the *F. nucleatum* subspecies polymorphum as the most significantly overrepresented species in OSCC tumors (163). This bacterium promotes cellular invasion and is particularly associated with OSCC cases in non-smokers and non-drinkers. Thus, it highlights its role in carcinogenesis independent of traditional risk factors (164). *F. nucleatum* can adhere to and invade epithelial and endothelial cells, disrupting β-catenin signaling, which leads to increased oncogene expression and enhanced cancer cell proliferation (165, 166). The oral microbiome also contributes to OSCC metastasis by modulating local immune responses and facilitating cancer cell metastasis to distant sites. For instance, *P. gingivalis*, a common oral bacterium, enhances the invasive potential of OSCC cells by activating MMPs and inducing EMT (162). In line with this, *F. nucleatum* induces oral cancer metastasis to the lungs through activation of intracellular autophagic mechanisms. Additionally, ECM remodeling was observed. *F. nucleatum* enhances metastasis via the production of outer membrane vesicles (OMVs), which are key drivers of N-cadherin upregulation and E-cadherin downregulation, thereby promoting metastatic progression (167). Oral bacteria are linked to resistance to chemotherapy and radiotherapy in OSCC treatment. *F. nucleatum*, for example, induces autophagy in OSCC cells, protecting them from the cytotoxic effects of chemotherapeutic drugs. This bacteria-driven chemoresistance can lead to treatment failure and poor outcomes for OSCC patients (166, 168). A study conducted by Liu et al. demonstrated that *F. nucleatum* altered autophagy in oral cancer by inducing autophagosome formation through the upregulation of LC3 and ATG7 gene expression. Notably, knockdown of ATG7 reversed the effect of *F. nucleatum*, rendering the cancer cells more sensitive to chemotherapy (169). In addition to the aforementioned bacteria, a plethora of other microbial species have been implicated in dysbiosis and the initiation and progression of oral cancer; however, a detailed discussion of these is beyond the scope of this review.

### 6.3 Metabolite production

The role of microbiome metabolite production in OSCC is an emerging area of research (170). Studies have shown that oral microbiota from OSCC patients, when exposed to ethanol, can produce carcinogenic levels of acetaldehyde, with higher levels observed in smokers (171). Through metabolomic analysis of saliva from Japanese OSCC patients, Oshima et al. identified potential biomarkers such as choline, branched-chain amino acids, urea, and 3-hydroxybutyric acid (172). This establishes an association between these metabolites and OSCC; however, direct evidence of their oncogenic potential remains limited, and further studies are required to validate their mechanistic role in tumor progression.

Similarly, research on South American OSCC patients revealed alterations in metabolic pathways such as the malate-aspartate shuttle, beta-alanine metabolism, and Warburg effect. Potential salivary biomarkers from this study include malic acid, maltose, protocatechuic acid, lactose, 2-ketoadipic acid, and catechol (173). The variations observed in metabolite profiles across these populations likely reflect underlying differences in genetic makeup, environmental exposures, diet, and microbiome composition, highlighting the importance of region-specific biomarker validation. Collectively, these findings expand our understanding of OSCC pathophysiology and underscore the promise of salivary metabolomics in early diagnosis, though further studies are needed to establish causal links and clinical utility.

### 6.4 Modulation of key signaling pathways

The oral microbiome influences key signaling pathways, contributing to the initiation and progression of OSCC. One such pathway is the E-cadherin/β-catenin signaling pathway, which is notably affected by *F. Nucleatum*. Activation of this pathway by *F. Nucleatum* triggers the EMT, enhancing oncogene expression and promoting cancer cell proliferation (101, 174). Moreover, microbial metabolites activate the PI3-K/Akt pathway, increasing cancer cell survival and resistance to apoptosis. The pro-inflammatory cytokine TNF-α further drives EMT by upregulating MMP-2 and MMP-9, processes regulated by NF-κB, Akt, and PI3-K signaling pathways. This results in enhanced invasion and metastasis of OSCC cells (115).

Another critical pathway disrupted in OSCC is the JAK/STAT pathway, with *P. gingivalis* playing a significant role by activating *STAT3*. This activation has several key implications (137, 175, 176). STAT3 activity inhibits immunological responses, allowing *P. gingivalis* and tumor cells to avoid detection and elimination. It also increases the transcription of genes involved in cell proliferation and survival, accelerating tumor growth. Furthermore, *STAT3* activation promotes the development of new blood vessels, encouraging tumor growth and metastasis. Both *F. nucleatum* and *P. gingivalis* are highly enriched in OSCC and may synergize with genetic and environmental factors to drive tumorigenesis. Understanding these mechanisms provides valuable insights for early detection, prognostic evaluation, and the development of targeted therapies for OSCC.

## 7 Emerging therapeutic strategies in OSCC

OSCC is an aggressive neoplasm, and traditional therapeutic modalities, such as surgery, chemotherapy, and radiation therapy, whether used alone or in combination, provide considerable challenges and notable side effects in practical applications (177). Contemporary therapeutic approaches involve using anticancer agents such as 5-fluorouracil, paclitaxel, cisplatin, and docetaxel monotherapies or synergistic combinations. Nonetheless, their toxicity to normal cells is significant when administered intravenously, as they exhibit non-specific distribution throughout the body, leading to considerable harm to healthy tissues and resulting in severe adverse reactions. The limitations of oral chemotherapy are further underscored by the low solubility, permeability, and inadequate bioavailability of these anticancer agents in physiological fluids (177). Consequently, advancing novel therapeutic regimens or refining existing methodologies is paramount for enhancing human health and ensuring survival in the face of oral cancer and its associated tissues. The dysfunction of OSCC pathways is crucial because it enables excessive cell growth, longevity, and metastasis (178). The management of OSCC also incorporates EGFR monoclonal antibodies and tyrosine kinase inhibitors (TKIs) (179). For recurrent and metastatic OSCC, cetuximab, a monoclonal antibody against the extracellular domain of EGFR, is given with chemotherapy and radiation (180). By halting tumor cell growth and spread, cetuximab helps in the deactivation of EGFR while stimulating the internalization and degradation of the receptor. Research shows that cetuximab administration improves outcomes during chemoradiotherapy, proven to be beneficial for progression-free survival (PFS) and overall survival (OS) among patients suffering from OSCC (181, 182). Noting the challenges associated with cetuximab resistance, which is frequently associated with mutations such as *EGFRvIII*, there is a considerable need for better patient stratification along different lines of treatment (183). Compared to cetuximab, nimotuzumab is an EGFR-targeting monoclonal antibody that has lower immunogenicity and thus has fewer side effects, especially skin toxicity (184). Using chemotherapy and radiotherapy along with nimotuzumab is effective in advanced OSCC, thus supporting its use as an alternative treatment (185). TKIs mark a milestone in the development of targeted cancer therapies as they reduce the damage expected with non-targeted approaches, especially the collateral injury inflicted on healthy tissues (186). TKIs like gefitinib, erlotinib, and afatinib block the downstream signaling required for tumor proliferation by binding to the intracellular domain of *EGFR*. Gefitinib was the first active EGFR-targeting-TKI and has shown moderate response rates during OSCC monotherapy, ranging from 1.4% to 10.6% (187). Despite these figures, gefitinib appears to have better clinical outcomes when combined with chemotherapy, achieving a median PFS of 4.8 months and an OS of 9.5 months (188). The response rates and survival with erlotinib combined with platinum and radiation therapy exceeded those of each therapy given alone (189, 190). Afatinib is a second-generation irreversible EGFR inhibitor aimed at both the wild-type and mutant EGFR, including the *EGFRvIII* mutation. It has shown comparable anti-tumor activity as cetuximab in phase II trials (191). Considering these results, afatinib may be an effective treatment option for EGFR-driven OSCC, particularly for non-responders to prior EGFR-directed therapies (179). The rapid increase in tumor cell population is associated with the development of new blood vessels that are critical sources of nutrients, oxygen, and growth factors needed for tumor maintenance and proliferation of tumor cells (192, 193). Angiogenesis is regulated by the VEGF pathway, which is crucial in OSCC. Monoclonal antibodies, particularly bevacizumab (194), along with multi-kinase inhibitors like sorafenib and vandetanib (195), target and block VEGF receptors, thus reducing angiogenesis and tumor growth. Bevacizumab, a humanized monoclonal antibody targeting VEGF-A, has demonstrated efficacy in many malignancies, including colorectal and non-small cell lung cancer. In OSCC, the combination of cetuximab, cisplatin, and radiation with Bevacizumab has enhanced outcomes and elevated progression-free and OS rates (196, 197). Nonetheless, particularly in combinatorial therapies, the danger of hemorrhage persists as a significant issue (198, 199). Sorafenib, a multi-kinase inhibitor, targets many receptors, including VEGFR1-3, PDGFR-β, and c-Kit, which facilitate tumor cell proliferation and angiogenesis (200). OSCC tumor cells demonstrate increased susceptibility to chemotherapy and radiation when administered sorafenib, suggesting its potential use in combination therapy (200, 201). Nonetheless, its application requires validation through direct efficacy assessments on OSCC in further clinical investigations (201).

Vandetanib is an inhibitor of the recently discovered tyrosine kinases, VEGFR-2 and EGFR, that demonstrated efficacy in preclinical investigations of OSCC. Vandetanib, in conjunction with cisplatin and radiation, has markedly diminished tumor burden, metastases, and microvessel density, indicating its potential application as both a chemopreventive and therapeutic drug for OSCC (202, 203). The survival, proliferation, and chemotherapy resistance of OSCC tumors are exacerbated by the often-disturbed PI3-K/Akt/mTOR signaling pathway. Preclinical and clinical research have identified a viable candidate in mTOR suppressor drugs, such as temsirolimus, which have demonstrated efficacy in anti-tumor preclinical models. Everolimus, a rapamycin derivative, has demonstrated efficacy when utilized in conjunction with other targeted medicines by augmenting radiosensitivity and tumor suppression (204). The observed clinical benefits were not significant, with certain trials indicating only moderate enhancements in PFS and OS (205). The therapeutic combination of mTOR inhibitors and EGFR inhibitors appears to be superior; nonetheless, the emergence of significant toxicities complicates patient management, necessitating heightened attention to detail. Mutations in *NOTCH1* that result in aberrant Notch signaling are crucial in the carcinogenesis and development of OSCC. Research is underway on gamma-secretase inhibitors to evaluate their potential in inhibiting the Notch signaling system. This strategy may prove advantageous in OSCC tumors marked by dysregulated Notch activity, hence providing viable alternatives for refractory OSCC instances that resist other treatment modalities (179).

The effective management and treatment of OSCC rely on several essential components, including CDKs and their function in regulating the cell cycle and cellular transcription (206). The overexpression of these kinases, particularly *PCNA* (proliferating cell nuclear antigen) and cyclin B1, correlates with increased OSCC metastasis and poor prognoses (207). Flavopiridol, ribociclib, and palbociclib are investigational drugs for cancer treatment due to their roles as CDK inhibitors. Although flavopiridol has demonstrated potential in preclinical investigations, its clinical use is significantly restricted due to side effects. Other CDK inhibitors, like palbociclib and ribociclib, have yielded inconsistent clinical outcomes in OSCC patients despite their established efficacy in advanced breast cancer (208). To date, few effective CDK inhibitors have been produced due to the highly homologous ATP-binding clefts on the CDK surface, complicating the targeting of a specific type. The creation of isoform-specific inhibitors is crucial (209).

Different approaches are also being explored. One therapeutic approach utilizes cyclooxygenase-2 (COX-2) inhibitors and endostatin (210). COX-2 exerts an oncogenic influence predominantly through the secretion of its pro-inflammatory mediators (211). COX-2 overexpression is frequently observed in several malignancies, especially in OSCC, and it significantly contributes to tumor proliferation, immune evasion, and angiogenesis. Celecoxib, a selective COX-2 inhibitor, has demonstrated efficacy in inhibiting the invasion, metastasis, and migration of OSCC cells. The concomitant administration of celecoxib and EGFR inhibitors, such as cetuximab, has demonstrated enhanced therapeutic efficacy due to the synergistic inhibition of COX-2 and EGFR (212–214), representing a significant treatment strategy for OSCC (179). Endostatin, an angiogenesis inhibitor, has demonstrated anti-tumor effects by causing apoptosis in endothelial cells and inhibiting blood vessel development. Endostatin has demonstrated the potential to diminish lymph node metastases and may serve as a viable adjunctive therapy for OSCC in conjunction with chemotherapy for refractory or treatment-resistant tumors (215, 216).

Systemic toxicity commonly accompanies traditional treatments like chemotherapy, and radiotherapy can cause significant side effects, including damage to healthy tissues. Limited therapeutic effect as some patients may not respond adequately to conventional treatments, leading to persistent or recurrent disease. Drug resistance is where cancer cells can develop resistance to chemotherapy drugs, reducing their effectiveness over time. Treatments can affect a patient’s quality of life and lead to complications such as dry mouth, inflammation of the mucous membranes, and restricted jaw movement. High-cost advanced treatments like targeted therapy and immunotherapy can be expensive for many patients. A delayed diagnosis may lead to poorer outcomes for patients diagnosed at later stages. Despite significant efforts in advancing immunotherapy for OSCC, the persistently low 5-year survival rate remains a major concern. This is largely due to the urgent need to better understand the genetic landscape (specifically identifying tumor-specific antigens) through the integration of sequencing technologies and bioinformatics, improve stratification scores, and gain a comprehensive insight into tumor-tumor, tumor-stromal, and microbiota interactions within the TME. Such knowledge is essential for developing personalized treatments that could improve overall survival and quality of life for patients. Furthermore, combination therapies are emerging as a promising approach to enhance survival and address the challenges in treating this stubborn disease. These limitations highlight the need for ongoing research and development of more effective, less toxic, and accessible treatment options for OSCC (10, 217, 218).

### 7.1 Potential therapeutic targets based on epigenetic modifications

Epigenetic modifications are critical in the development of OSCC and display potential targets for therapy and biomarkers. DNA methylation inhibitors like 5-Azacytidine and Decitabine can reverse the hypermethylation of TSG, while histone deacetylase (HDAC) inhibitors such as Trichostatin A and Vorinostat help restore normal histone acetylation (219). On the other hand, miRNA-based therapies, including synthetic miRNA mimics and antagomiRs, show promise in reactivating tumor-suppressing miRNAs or blocking oncogenic ones (220, 221). Recent studies indicate that combination therapies, including HDAC inhibitors and cisplatin, enhance chemosensitivity in OSCC cells. Novel targets, including DOT1L (Disruptor of Telomeric Silencing 1-Like), are linked to cancer stem cell invasion and cisplatin resistance, whereas selective mTOR inhibitors in conjunction with HDAC inhibitors are under investigation (222). Salivary miRNAs, particularly miR-423-5p, have emerged as potential prognostic biomarkers, as elevated levels in preoperative saliva are associated with reduced disease-free survival in OSCC patients (220, 223).

### 7.2 Immunotherapy in OSCC: current progress, challenges, and future directions

A significant advancement in cancer therapy has been made by utilizing the immune system to identify and eliminate tumors. Cancer immunotherapeutic strategies encompass vaccinations, adoptive T-cell treatment, and immune checkpoint blockade therapy (224). Given the essential role that the immune system plays in the progression and management of OSCC, early strategies aimed at exploiting immunity to mitigate cancer garnered widespread attention. A groundbreaking advancement in this field was the U.S. Food and Drug Administration (FDA) approval of T-VEC (Talimogene) in 2015, an HSV-1-based oncolytic virus armed with GM-CSF (Granulocyte-Macrophage Colony-Stimulating Factor), for the treatment of melanoma (225). Since then, T-VEC has been investigated for use in breast cancer (226), pancreatic cancer, and HNSCC (227). One year later, in 2016, the FDA approved another transformative innovation, immune checkpoint inhibitors (ICI), for the treatment of metastatic and inoperable HNSCC patients. This transformative modality demonstrated robust efficacy in HNSCC patients, paving the way for an effective treatment for this heterogeneous solid tumor (228, 229). The efficacy of ICIs is limited to patients possessing particular biomarkers and is contingent upon the expression levels of immune checkpoints like PD-L1 (230, 231). Mechanistically, anti-PD-1/PD-L1 antibodies (also known as PD-1/PD-L1 inhibitors) work by restoring T-cell activity within the TME. In the KEYNOTE-048, a phase III randomized clinical trial, pembrolizumab (an anti-PD-1 monoclonal antibody) was evaluated as monotherapy and in combination with chemotherapy, compared to cetuximab with chemotherapy (230). The results demonstrated that pembrolizumab combined with chemotherapy significantly improved overall survival, surpassing cetuximab plus chemotherapy in PD-L1-positive patients with HNSCC, including those with OSCC (230). This study established that the combination of pembrolizumab with platinum and 5-fluorouracil is both safe and effective and is now considered an appropriate first-line treatment for this patient group. However, treatment responses remain largely confined to biomarker-defined subgroups, highlighting the importance of predictive markers such as tumor mutational burden (TMB). Understanding the TME, the dynamics of immune-infiltrating cells, the genetic landscape, and immune profiling is crucial for tailoring treatments for cancer patients, including those with OSCC. Numerous clinical trials investigating a wide range of immunotherapeutic treatments have been completed, with many still ongoing. **Table 1** provides a comparison of different immunotherapeutic modalities, highlighting their pros and cons. Harrington et al. conducted a multicenter phase Ib clinical trial involving 36 patients with recurrent/metastatic squamous cell carcinoma of the head and neck. The trial aimed to investigate the potential of enhancing treatment with the oncolytic vaccine, T-VEC, in combination with ICI, pembrolizumab, following a low response to monotherapy with ICI treatment (ClinicalTrials.gov Identifier: NCT02626000) (232). This combination was shown to be safe compared to treatment with pembrolizumab alone. However, the trial was not advanced to phase III as initially planned (232). Mell et al. conducted a phase I clinical trial to evaluate the safety profile of GL-ONC1, a recombinant vaccinia virus that encodes three transgenes: Ruc-GFP, β-glucuronidase, and β-galactosidase. This double-stranded DNA platform is regarded as highly promising for its capacity to selectively lyse tumor cells while preserving normal tissue (233). Its short, well-defined life cycle, efficient cell-to-cell spreading, and ample use as a smallpox vaccine contribute to its high safety profile (234, 235). This Phase I study was conducted to determine the maximum tolerated dose (MTD), identify dose-limiting toxicities, and recommend a dose for a Phase II study of GL-ONC1 when administered intravenously in combination with chemoradiotherapy for primary non-metastatic HNSCC patients. GL-ONC1 was found to be safe and well-tolerated when given intravenously and demonstrated improvements in OS and PFS (233). A phase II trial is, however, warranted for this oncolytic vaccine platform (233). Nevertheless, no FDA-approved oncolytic virus has been approved exclusively for OSCC due to the disease’s complexity and the limited data from preclinical research. However, oncolytic vaccines are gaining attention, with recent research and preclinical studies in this area. In a commentary, Raja emphasized the potential of oncolytic vaccines to revolutionize OSCC treatment and improve patients’ lives in the future (236). In line with this, preclinical trials investigating the efficacy of oncolytic viruses in OSCC have been conducted. Gohara et al. demonstrated that OBP-301, in combination with radiotherapy, is a novel therapy effective in treating radioresistant OSCC (237). This vaccine, which uses an adenoviral backbone encoding the human telomerase reverse transcriptase (hTERT) promoter (238)—exclusively expressed in cancer cells—shows promise in enhancing treatment efficacy. The observed effects are attributed to the STAT3-Bcl-Xl axis, which regulates autophagy and apoptosis, thereby improving radiosensitivity (237). OBP-301 warrants further investigation in refractory OSCC patients and those resistant to standard therapies. Despite advancements in understanding the OSCC tumor immune microenvironment and the promising outcomes of various immunotherapeutic strategies, significant challenges remain in translating these findings into practical clinical applications (239). The process of selecting and evaluating the effectiveness of immunotherapy, whether used alone or in combination with other treatments, requires improved methodologies to assess synergy and minimize side effects (240). Moreover, improving long-term survival with multi-agent immunotherapy strategies requires a careful balance between efficacy and toxicity to provide lasting benefits (239). Furthermore, regulatory obstacles pose significant challenges in approving and clinical application of innovative immunotherapies, consequently hindering their broader adoption (240).

**Table 1:** Comparison of various immunotherapeutic modalities: mechanistic action, advantages, and disadvantages.

Immunotherapy	Mechanism of action	Clinical use	Advantages	Disadvantages
Checkpoint Inhibitors	Block inhibitory pathways (e.g., PD-1, CTLA-4) to enhance T-cell activation and anti-tumor immunity.	It is used in various cancers, including OSCC, melanoma, and lung cancer.	Effective in cancers with immune evasion; potentially long-lasting immunity.	Immune-related adverse reactions (e.g., colitis, hepatitis, hypothyroidism). Not effective in all patients.
Targeted Monoclonal Antibodies	Bind to specific tumor-associated antigens (e.g., EGFR, MUC-1) to inhibit tumor growth or enhance immune response.	EGFR-targeted therapies (e.g., cetuximab) in head and neck cancers.	Selective targeting of cancer cells can be combined with other treatments like chemotherapy or radiotherapy.	May not work in all patients due to mutations or tumor heterogeneity; can cause allergic reactions or infusion-related issues.
Adoptive Cell Transfer (ACT)	T-cells are extracted, expanded, and reintroduced into the patient, often engineered with tumor-specific receptors (e.g., CAR-T).	Used for melanoma, cervical cancer, and potentially OSCC with HPV-specific T-cells.	Highly personalized treatment; can lead to significant tumor regression.	Complex production process; potential for cytokine release syndrome (CRS); limited by T-cell exhaustion or tumor heterogeneity.
Cancer Vaccines	Stimulate the immune system to recognize and attack cancer cells by using tumor antigens or whole tumor cells.	FDA-approved vaccines for melanoma (e.g., BCG vaccine) and research for OSCC and other cancers.	Can generate long-lived immunity; minimal toxicity; can be combined with other therapies.	Expensive; slow immune response; limited effectiveness in rapidly growing tumors.
Cytokine Immunotherapy	Enhance immune system activity by using molecules like IL-2 and IFN-α to boost immune cells such as NK cells.	Used in melanoma and renal cell carcinoma; investigational use in OSCC and other cancers.	Boosts immune response; can increase tumor-infiltrating lymphocytes (TILs).	High toxicity (fatigue, diarrhea, pancytopenia); pleiotropic effects that can harm normal cells.

ACT, Adoptive Cell Transfer; BCG vaccine, Bacillus Calmette-Guérin vaccine; CAR-T, Chimeric Antigen Receptor T-cell therapy; CRS, Cytokine Release Syndrome; CTLA-4, Cytotoxic T-Lymphocyte-Associated Protein 4; EGFR, Epidermal Growth Factor Receptor; HPV-specific T-cells, Human Papillomavirus-specific T-cells; IFN-α, Interferon-alpha; IL-2, Interleukin-2; MUC-1, Mucin-1; NK cells, Natural Killer cells; OSCC, Oral Squamous Cell Carcinoma; PD-1, Programmed Cell Death Protein 1; TILs, Tumor-Infiltrating Lymphocytes.

### 7.3 Potential therapeutic targets

The recent identification of several possible therapeutic targets for OSCC presents novel treatment avenues for this complex cancer. EGFR inhibition: Previous research indicated that OSCC cells frequently demonstrate an overexpression of EGFR. Consequently, inhibiting the receptors is a viable treatment alternative, as the antibodies, cetuximab, have previously received approval for use. Furthermore, it was shown that gefitinib, an EGFR TKI, markedly enhances the efficacy of cisplatin against OSCC cells. Research demonstrates that gefitinib reduces the activity of EGFR and the downstream signaling pathways, ERK and Akt, in a dose-dependent manner (241). Recent research indicated that EGFR amplification is prevalent among younger patients diagnosed with OSCC, suggesting that targeting these receptors may be advantageous for this group (242). Vascular Endothelial Growth Factor Receptor (VEGFR) Inhibition: The signaling mediated by the VEGFR is essential for the progression of OSCC. Research revealed further anti-proliferative effects in OSCC cells when the VEGFR inhibitor vatalanib was administered in conjunction with other EGFR inhibitors. The most compelling finding arose from the EGFR-knockout OSCC cells, which exhibited elevated expression of VEGFR2, suggesting a potential compensatory mechanism (105). Nanoparticle-based drug delivery: Nanoparticles have been utilized in the treatment of OSCC, demonstrating enhanced drug bioavailability and selective tumor targeting, which results in improved biocompatibility, efficacy, and reduced systemic toxicity and side effects associated with oral cancer therapy. A range of nanoparticles has been examined for their efficacy against OSCC, including:

- Polymeric nanoparticles: Polymers of both natural and synthetic sources, such as polylactic acid, polyglycolic acid, polysaccharides, polycaprolactone, and polyethylene glycol, are used to entrap or adhere to the therapeutic agent (243).
- Liposomes: Artificial vesicles made of a phospholipid bilayer capable of encapsulating therapeutic agents of both hydrophilic and hydrophobic nature to the targeted site of action. Since the frugs loaded on liposomes can be identified by reticuloendothelial systems, polyethylene glycol can be used to cover the liposomal surface (244).
- Gold nanoparticles (AuNPs): They are characterized by good biocompatibility and high permeability, thus, AuNPs have been used in many biomedical fields. AuNPs have many geometric shapes, such as nano shells, nanorods, nanosphere, and nanocages, making them effective carriers for the therapeutic agents to the target site (243).
- Hydrogel: A three-dimensional porous material that is injected directly into the site of injury, thus avoiding the need for intravenous administration of small NPs and providing the advantage of controlled release of the drug (243, 245).

Combination therapy has been shown to enhance outcomes for individuals with OSCC by integrating multiple treatment modalities. The “EXTREME” regimen, utilized as the primary treatment for recurrent HNSCC cases, integrates cetuximab with platinum and 5-FU, followed by maintenance cetuximab. Alternative combinations, including taxanes with cisplatin and cetuximab, have demonstrated considerable therapeutic efficacy (246). Recent endeavors have focused on evaluating the effectiveness of augmenting epigenetic modifiers, such as 5-aza-2’-deoxycytidine (5-AZA-2CdR), in conjunction with conventional chemotherapy drugs like cisplatin. This approach alters the methylation patterns of critical DNA repair and drug-resistance genes, potentially enhancing chemotherapy efficacy (247, 248). These targeted and combination therapeutic approaches exhibit promising advancements in OSCC treatment outcomes; nevertheless, extensive research is necessary to enhance their efficacy and reduce potentially harmful effects.

## 8 Personalized medicine in the treatment of OSCC

Recently emerging approaches in personalized medicine demonstrate encouraging advancements in biomarker identification and targeted therapeutics. Recent studies demonstrated the potential of minimally invasive biomarkers, including circulating tumor cells (CTCs) and molecular targets, in facilitating early diagnosis and monitoring therapy responses (10). Much research concentrated on targeting specific cellular receptors, such as EGFR. A previous study indicated that younger OSCC patients (below 50 years of age) had EGFR amplification and increased RNA levels. This discovery indicates that EGFR amplification will serve as a significant therapeutic target for this patient subgroup (249). The advancement and progression of OSCC are significantly affected by epigenetic regulation, serving as a crucial mechanism for the formulation of epigenetic-targeted therapies, including DNA methyltransferase (DNMT) inhibitors and HDAC inhibitors, which mitigate aberrant gene expression and address drug resistance (250). Personalized medicine may also integrate advanced imaging techniques, including confocal microscopy (CM) and optical coherence tomography (OCT), which assist in evaluating suspicious oral lesions and various histological stages of OSCC. These methods are non-invasive, facilitating early tumor detection and precise treatment planning (251, 252). Combined therapy targeting several pathways has also been investigated. They evaluated the possibility of EGFR and Cortactin co-expression as predictors for dual-target treatment in OSCC. The study determined that the co-expression of EGFR and Cortactin was associated with poor survival rates, suggesting the potential for dual protein targeting in advanced OSCC cases (253). Research continues to be conducted to identify reliable biomarkers and effective therapeutic combinations. We anticipate that the integration of multi-omics data with artificial intelligence will enhance the complexity of data processing, resulting in improved discovery of novel therapy targets and prediction of treatment results. Nevertheless, the consistent implementation of biomarker testing and personalized treatment remains a difficulty yet to be resolved for clinical applications.

### 8.1 Transformative role of personalized medicine in OSCC immunotherapy

Molecular profiling encompasses the analysis of tumors at the cellular level, employing genomic, proteomic, and metabolomic approaches. These technologies can help discover targetable immunotherapy genetic aberrations along with their associated prognostic markers. Biomarker characterization, such as the expression of PD-L1 and tumor mutational burden (TMB), may enrich the patient populations for whom immunotherapy is likely to be successful. **Table 2** presents the types of biomarkers and their clinical relevance in OSCC. The administration of anti-PD-L1 antibody therapy has been crucial for the management of OSCC (283, 284). Recent studies have examined PD-L1 in specimens from patients who received immunotherapy to identify cases likely to respond to the treatment. For example, Emancipator et al. (285) determined that a “combined positive score”, defined as the ratio of PD-L1 expressing cells (both cancerous and immune) to the total number of viable cancer cells, multiplied by 100, is effective in assessing the response to pembrolizumab (285). In other investigations, PD-L1 expression was substantially linked with the response to the anti-PD-L1 antibody durvalumab (286, 287). These investigations established that a threshold of 25% of cancer cells exhibiting PD-L1 staining is considered appropriate for evaluating patients’ responses to durvalumab immunotherapy (286, 287). Some work has been conducted on the predictive significance of tumor mutational burden (TMB) in relation to immunotherapy, specifically with pembrolizumab. Cristescu et al. indicated that the conjunction of elevated TMB and T cell inflamed gene expression profiles correlates with improved survival outcomes in patients who received pembrolizumab (288). A tumor mutational burden of 10 mutations per megabase is frequently the criterion for initiating treatment with pembrolizumab, although its widespread application remains controversial. Strickler et al. consistently account for the potentially confounding influence of prior cytotoxic chemotherapy, which may elevate TMB, rather than formulating treatment recommendations solely based on this variable (289). Moreover, personalized medicine enables the development of interventions tailored to the unique characteristics of a patient’s tumor, potentially enhancing efficacy. For several cancers, including OSCC, a reliable approach for assessing tumor TILs in H&E-stained sections has been established (290). A study by Almangush et al. demonstrated the significance of assessing stromal TILs to forecast overall survival, disease-specific survival, and disease-free survival rates in patients with OSCC, particularly in early-stage oral tongue cancer cases. Such simple methods of TIL evaluation should be studied in validation studies with larger sample sizes and could improve the evaluation of the impact of immunotherapy on the patients (291). Such simple methods of TIL evaluation should be studied in validation studies with larger sample sizes and could improve evaluating the impact of immunotherapy on the patients. By understanding the molecular profile of a patient’s tumor, clinicians can design combination therapies that enhance the effectiveness of immunotherapy. For example, combining checkpoint inhibitors with other treatments like chemotherapy or radiotherapy. Personalized medicine has the potential to improve patient outcomes by providing more precise and effective treatments paving the way for more effective and individualized treatment strategies in OSCC, offering hope for better outcomes and reduced side effects (292–294).

**Table 2:** Types of biomarkers and their clinical relevance in OSCC.

Biomarker	Mechanism of action of action	Clinical utility	Limitations	Prognostic Relevance in OSCC	References
p53 Gene	Tumor suppressor gene regulating apoptosis, cell cycle, and DNA damage response.	Potential target for gene therapy.	Mutations may be heterogeneous.	Overexpression is linked to progression and poor survival.	(254–256)
EGFR	Receptor tyrosine kinase (RTK) promotes cell proliferation.	Therapeutic target for monoclonal antibodies and TKIs. Improved survival with targeted therapies combined with chemo/radiotherapy	Not all overexpressing patients respond to therapies.	Overexpression is associated with poor prognosis and aggressive behavior.	(254, 257, 258)
Cyclin D1	Regulates G1 to S phase cell cycle progression.	Immunohistochemical assessment provides prognostic information.	Overexpression alone may not fully predict outcomes.	Overexpression correlates with tumor aggressiveness and poor prognosis.	(254, 259, 260)
MMPs	Enzymes degrading extracellular matrix (ECM), facilitating invasion and metastasis.	Levels can inform on invasiveness.	Broad substrate specificity can complicate interpretation.	Elevated levels are linked to increased metastatic potential and poor prognosis.	(261)
miR-21	MicroRNA promoting oncogenesis. Regulates gene expression post-transcriptionally	Emerging as a non-invasive salivary biomarker.	Detection requires specialized techniques.	Overexpression is linked to tumor growth, invasion, and therapy resistance.	(262)
miR-375	MicroRNA is involved in tumor suppression.	Potential prognostic marker.	Detection is complex and not yet routine.	Downregulation is associated with advanced stages and poorer outcomes.	(263)
Ki-67	Nuclear protein associated with cellular proliferation.	It is commonly used to assess tumor proliferation rates.	It may not distinguish benign from malignant proliferation.	High expression correlates with increased aggressiveness and poor prognosis.	(264, 265)
Survivin	Inhibitor of apoptosis protein. It prevents cell death and promotes cell division.	Potential target for novel therapies.	Role in normal tissues can complicate its use as a specific marker.	Overexpression is linked to therapy resistance and reduced survival.	(44)
Podoplanin	Glycoprotein involved in cell migration and tumor invasion.	Utilized as a marker for tumor invasiveness and lymph angiogenesis.	Expression in some normal tissues may limit specificity.	Expression associated with lymph node metastasis and decreased survival.	(266)
PD-L1	Immune checkpoint protein inhibiting T-cell activation.	Target for immune checkpoint inhibitors.	Expression levels can be heterogeneous.	Expression is associated with immune evasion and may predict response to immunotherapy.	(154, 230, 267, 268)
p16	Tumor suppressor protein regulates the cell cycle.	Used as a surrogate marker for HPV-associated OSCC. Aids in prognosis and treatment planning in HPV+ OSCC.	Prognostic value is primarily significant in HPV-related cases.	Overexpression associated with better prognosis in HPV+ OSCC.	(269, 270)
VEGF	Promotes angiogenesis	Potential target for anti-angiogenic therapies.	Targeting VEGF can lead to adverse effects.	Elevated levels correlate with increased angiogenesis and poor prognosis.	(271)
TFRC	Mediates cellular iron uptake.	Proposed as a novel prognostic biomarker and potential therapeutic target.	Expression in immune cells and bone marrow may complicate its use.	Overexpression is linked to increased proliferation and poor prognosis.	(272)
NCBP2	Involved in mRNA processing and stability. Influences gene expression.	Suggested as a novel prognostic biomarker and potential target.	Lack of extensive research.	Overexpression is associated with poor prognosis.	(272)
MRGs	Regulate mitochondrial autophagy.	Proposed prognostic model based on MRG expression.	The complexity of autophagy pathways may complicate interpretation.	Altered expression associated with OSCC progression and patient outcomes.	(273)
C-reactive protein/albumin ratio (CAR)	Combines CRP and albumin levels. Assesses inflammation and nutritional status.	Simple, cost-effective, and widely used. Used as a systemic inflammatory marker.	Non-specific to OSCC.	Elevated CAR is linked to poor prognosis.	(274)
KRT17	Structural protein in epithelial cells used in tumor differentiation and prognosis assessment	Tissue biomarker with potential for histological evaluation.	Requires immunohistochemical analysis.	Promotes OSCC progression.	(275)
CD44	Glycoprotein is involved in cell adhesion.	Marker for cancer stem cells. Evaluated in targeted therapies.	Not specific to OSCC.	High expression is linked to EMT and poor survival.	(276)
ET-1	Peptide is involved in angiogenesis and metastasis.	Explored in ET receptor-targeted therapies.	Involved in multiple cancers.	Enhances OSCC progression.	(277)
CD59	Complement regulatory protein inhibits complement-mediated lysis.	Protects cells from immune attack. Investigated for immune-targeted therapies.	It may facilitate immune evasion.	Reduced expression linked to OSCC transformation.	(278)
Gal-1/Gal-3	Glycoproteins involved in apoptosis and adhesion.	Potential biomarker for tumor aggression.	The role varies in different tumors.	Overexpression linked to OSCC progression.	(279)
SCC Ag	Tumor-derived protein involved in immune evasion.	Useful for monitoring recurrence and clinical prognosis.	Elevated in other SCCs.	Increased levels indicate advanced OSCC.	(280)
Cancer antigen 125 (CA-125)	Glycoprotein produced in epithelial structures	Used in tumor diagnosis and prognosis assessment.	Lacks specificity, as elevated levels can occur in benign conditions	Elevated in OSCC, especially in saliva	(281, 282)

CA-125, Cancer Antigen 125; CAR, C-reactive Protein/Albumin Ratio; CD44, Cluster of Differentiation 44; CD59, Cluster of Differentiation 59; EGFR, Epidermal Growth Factor Receptor; EMT, Epithelial-Mesenchymal Transition; Gal-1/Gal-3, Galectin-1/Galectin-3; HPV, Human Papillomavirus; Ki-67, Marker of Proliferation Ki-67; KRT17, Keratin 17; MMPs, Matrix Metalloproteinases; miR-21, MicroRNA-21; miR-375, MicroRNA-375; MRGs, Metabolism-Related Genes; NCBP2, Nuclear Cap-Binding Protein Subunit 2; OSCC, Oral Squamous Cell Carcinoma; p16, Cyclin-Dependent Kinase Inhibitor 2A; PD-L1, Programmed Death-Ligand 1; SCC Ag, Squamous Cell Carcinoma Antigen; TFRC, Transferrin Receptor; TKIs, Tyrosine Kinase Inhibitors; VEGF, Vascular Endothelial Growth Factor.

## 9 Challenges in translating molecular research into clinical applications for the management of OSCC

Integrating therapeutic science into clinical care for OSCC has proven to be a complex endeavor even with the advancements made in molecular oncology. Implementing novel drugs and biomarker testing into standard clinical practice is hindered by several scientific, logistical, and patient-focused challenges that restrict the adoption of precision medicine in OSCC treatment. Significant obstacles stem from tumor heterogeneity, including genetic, epigenetic, and molecular differences within and between individuals (295, 296).

This variety impedes the establishment of common therapeutic markers and contributes to discrepancies in treatment response. For instance, some regions within a singular tumor may contain different abundances of cancer-associated fibroblasts (CAFs), immune cells, and ECM constituents, which lead to regional differences in treatment efficacy (297). Such diversity requires customized therapeutic approaches that consider specific features of individual tumors’ microenvironments rather than generic approaches (295). Moreover, the implication of the TME on OSCC growth and treatment resistance is another barrier. Composed of an intricate network of immune cells, fibroblasts, ECM building blocks, and hypoxic areas, it regulates tumor behavior through a complex interplay. The identification and validation of effective biomarkers for TME-directed therapies remain an important focus of the research. Other authors have shown markers like fibroblast activation protein (FAP) for the possible evidence of activity of CAF in OSCC that may help guide treatment aimed at altering the interactions between stromal tissue and the tumor (298). The possibility of identifying these biomarkers in patients, which is more challenging, may enhance the accuracy of treatment by enabling the monitoring and modification of treatment plans based on the changes within the TME in real-time (295). Another challenge that negatively impacts the efficacy of immune checkpoint inhibitors and EGFR inhibitors is resistance to targeted therapy. There may be intrinsic or acquired resistance because of tumor mutations, activation of compensatory survival pathways, or the existence of TME that promotes evasion of the immune response.

The latter introduces further resistance by recruiting immunosuppressive cell types, such as MDSCs, and changing cellular metabolism (295). There are efforts to propose a combination therapy directed at tumor-supporting pathways and the environment that supports these pathways. The attempt to improve therapy effectiveness already includes MDSC-targeting strategies and immune checkpoint blockade in combination with CAF-disrupting agents (299–302).

Targeted molecular therapies represent a step forward, but there are still numerous risks regarding their side effects (303). Using immune checkpoint drugs, EGFR inhibitors (304), and kinase-targeted medicines (305) can have grave consequences for side effects, which might diminish patients’ quality of life and compliance. For instance, cetuximab, an EGFR-targeted medication, has been associated with severe skin toxicity, while erlotinib, a TKI targeting EGFR, inhibits the effector’s action, which leads to the development of drug resistance, and while doing so, immune checkpoint inhibitors could bring about immunological side effects (304). Other contraindications are gastrointestinal disorders like diarrhea, the probable sequel of increased epithelial cell turnover and mucosal damage, fatigue, nausea, vomiting, and changes in electrolyte balance (306, 307). Managing these toxicities requires carefully balancing and enhancing therapeutic effectiveness with maintaining the patient’s well-being, which is especially challenging in OSCC patients who frequently suffer from significant functional and cosmetic deficits because of tumor burden and previous treatments. A lack of standardized treatment protocols that integrate the newest advances in molecular-targeted therapy is yet another issue. Cetuximab, an EGFR inhibitor, is utilized in conjunction with chemotherapy (308), radiation (309), and ICIs such as anti-PDL-1 (310). However, optimal administration methods, sequencing of treatments, and patient selection criteria remain under investigation (311). Clinical decision-making is further complex, and the integration of new treatment options into standard practice is constrained for these reasons. A holistic strategy, encompassing molecular analysis, biomarker-driven patient categorization, resistance-targeted combination therapy, and supportive care for treatment-related toxicity, is essential. The implementation of novel research into effective and customized OSCC treatment will be enhanced by establishing standardized treatment protocols grounded in developing clinical evidence.

## 10 Recent highlights from key studies

Despite the recent breakthroughs in immunotherapeutic treatments and their potential enhanced effects when combined with other treatment modalities or conventional therapies, traditional approaches such as surgery, radiotherapy, chemotherapy, or chemoradiotherapy remain the gold standard for patients with OSCC. **Table 3** summarizes selected completed and ongoing clinical trials investigating treatment strategies in OSCC. A recent clinical trial involving patients with high-risk recurrent OSCC and extranodal extension greater than 2 mm showed improved prognosis in those who received postoperative treatment with either radiotherapy or chemoradiotherapy (320). Additionally, conventional therapies have been investigated in clinical trials in combination with immunotherapy to enhance the efficacy of immune-based treatments and improve the 5-year survival rate in early-stage, advanced, invasive, and non-metastatic oral squamous cell carcinoma (OSCC). In a two-arm, open-label phase II clinical trial (NCT05821452), Chen et al. hypothesized that combining chemotherapy with camrelizumab, an anti-PD-1 antibody, may enhance the immune response in locally advanced unresectable esophageal squamous carcinoma. Chemotherapy is proposed to prime the immune system by activating tumor antigens, thereby reducing tumor immune evasion (321). This could result in a stronger immune response, reduced side effects, and allow for lower doses and shorter treatment durations (321). Ultimately, this approach aims to shrink the tumor and enable surgical resection in cases initially deemed inoperable. The trial is expected to conclude by the end of May 2026. In line with this, a randomized two-arm phase II clinical trial by Liu et al. investigated the efficacy of combining neoadjuvant immunotherapy (camrelizumab, an anti-PD-1 inhibitor) with or without docetaxel-cisplatin-5-fluorouracil (TPF) chemotherapy in patients with resectable locally advanced OSCC (316). The study reported that the combination therapy achieved significantly improved outcomes, including a major pathological response rate of 76.4% and a 2-year event-free survival rate of 91.2%, both of which were markedly higher than those observed with camrelizumab monotherapy, which yielded survival rates of 14.7% and 52.9%, respectively (316). These results suggest that neoadjuvant immunotherapy combined with chemotherapy is both feasible and safe in patients with resectable OSCC (322). In the context of neoadjuvant therapy, a pilot clinical trial for early-stage OSCC investigated the use of imiquimod, a toll-like receptor 7 agonist. The treatment resulted in a 50 percent reduction in tumor cell count in 60 percent of patients post-treatment, along with an immune-related pathological response in the same proportion of patients (322). This tumor reduction was attributed to increased activity of functional helper T cells and cytotoxic T cells. These findings suggest that neoadjuvant immunotherapy may represent a promising strategy for managing OSCC at early stages (322). Collectively, these recent clinical studies highlight the growing potential of immunotherapy, particularly in the neoadjuvant setting and in combination with other modalities, as a promising strategy for managing OSCC. While conventional treatments remain the cornerstone of care, emerging evidence supports the integration of immunotherapeutic agents to enhance response rates, improve survival outcomes, and potentially enable surgical intervention in previously unresectable cases. Further validation through large, multicenter clinical trials is essential. Furthermore, identifying reliable predictive biomarkers is a critical step toward realizing personalized immunotherapy strategies for OS.

**Table 3:** OSCC therapies in Clinical trials.

Type of therapy	Status/Country	Route of administration	Target population	Clinical phase and last year update	Combination treatment	Main findings	NCT identifier	Ref.
Immune checkpoints inhibitors (Neoadjuvant use prior to surgery)	Completed/ China, Shanghai	i.v. (camrelizumab)	Locally advanced resectable oral squamous cell carcinoma	Phase I (2023)	Apatinib (VEGRR2) Orally	Combining anti-PD-1 therapy (camrelizumab) with a VEGFR inhibitor as a neoadjuvant treatment is safe and well tolerated, achieving a major pathological response (MPR) in 40% of patients, characterized by high CD4^+^ T cell infiltration and low expression levels of CD31 and α-SMA	NCT04393506	(312)
NRC-2694-A (EGFR tyrosine kinase inhibitor)	Active, not recruiting/United states & India	PO	Recurrent or metastatic HNSCC	Phase II (2025)	Intervention + Paclitaxel	Trial is currently ongoing. Preliminary results of 19 out of 46 patients ORR provided sufficient evidence to reject the null hypothesis in support of alternative hypothesis.	NCT05283226	(313)
Dose-dense TPF Induction Chemotherapy	Completed/NA	i.v.	locally advanced head and neck cancer	Phase II (2020)	Intervention (TPF) is a combination therapy	The induction regimen proved to be active and well tolerated, with an RR of 89.6%, including a CR rate of 31% and PR rate of 58.6%. The 3-year OS was 54.3%, and PFS was 34.3%	NCT04397341	(314)
Neoadjuvant chemoimmunotherapy (Toripalimab+ Docetaxel+ Cisplatin)	Not yet recruiting/China, Guangdong	i.v.	Locally Advanced Oral and Oropharyngeal Cancer	Phase II (2025)	SBRT (Stereotactic Body Radiotherapy)	Inconclusive	NCT06861712	NA
Neoadjuvant Chemotherapy (Taxane, Cisplatin, and 5-FU)	Completed/ Indonesia, Jakarta	i.v.	Locally advanced oral squamous cell carcinoma	Phase III (2019)	Melatonin (20 mg Orally)	Adding melatonin to neoadjuvant chemotherapy reduced the levels of miR-210 and CD44, which are markers of hypoxia, cancer stemness, therapy resistance, angiogenesis, and recurrence; however, the reduction was not statistically significant compared to the placebo cohort	NCT04137627	(315)
Neoadjuvant immunotherapy (camrelizumab)	China, Wuhan/Completed	i.v.	Locally advanced oral squamous cell carcinoma	Phase II (2025)	TPF (docetaxel-cisplatin-5-fluorouracil)	MPR and 2-year disease-free survival were higher in the combination arm (camrelizumab + TPF) compared to camrelizumab alone. This trial indicates that neoadjuvant immunotherapy is both safe and feasible for treating OSCC patients	NCT04649476	(316)
Toripalimab (anti-PD-1)	Completed/China	i.v.	Locally advanced OSCC	Phase I (2023)	Intervention + Paclitaxel/​Cisplatin + Radical surgery + Post-operative radiotherapy/chemoradiotherapy	The treatment was well tolerated, with a low incidence of grade 3–4 adverse events and a high MPR rate of 60%	NCT04473716	(317)
Avelumab	Active, not recruiting/France	i.v.	Locally advanced SCCHN	Phase III (2025)	Intervention + Cetuximab + IMRT	The safety phase demonstrated that combination therapy with avelumab and RT-cetuximab was well tolerated in patients with locally advanced squamous cell carcinoma	NCT02999087	(318)
Nivolumab	Completed	IV	Locoregional OCSCC	Phase II (2021)	Intervention + definitive surgical resection	Neoadjuvant nivolumab therapy showed a well-tolerated safety profile and overall RR of 33%	NCT03021993	(319)
Radiotherapy and/​or chemotherapy(Cisplatin)	Unknown/Taiwan	i.v.	Primary Oral Squamous Cell Carcinoma	Phase II (2016)	NSAID (Celecoxib) cyclooxygenase-2 (COX-2) inhibitor	Inconclusive	NCT02739204	NA
Neoadjuvant combination of immunotherapy (Tislelizumab, anti-PD-1) +targeted therapy (Anti-EGFR) + Chemotherapy (cisplatin and nab-paclitaxel) followed by salvage surgery and adjuvant therapy	China/Shanghai	i.v.	Resectable, Locally Recurrent Oral and Oropharyngeal Squamous Cell Carcinoma	Phase II (2024)	Chemotherapy (cisplatin and nab-paclitaxel)	Inconclusive	NCT06728618	NA
TPF induction chemotherapy	Terminated/China	IV	cN2 OSCC patients	Phase II (2023)	Intervention + Surgery + Radiotherapy	Inconclusive	NCT02285543	NA
Nisin (apoptogenic bacteriocin)	Recruiting/United States	PO	OSCC	I/II (2025)	Intervention + Surgical resection	Inconclusive	NCT06097468	NA

i.v., intravenous; PO, oral; TPF, docetaxel, cisplatin, and 5-fluorouracil; RR, Response rate; CR, complete response; PR, partial response; OS, Overall survival; PFS, progression-free survival; MPR, Major pathological response; IMRT, intensity modulated radiotherapy; OCSCC, Oral cavity squamous cell carcinoma; SCCHN, Squamous cell carcinoma of head and neck; HNSCC, head and neck squamous cell carcinoma.

## 11 Conclusion: challenges and future directions in OSCC research

Notwithstanding considerable endeavors by researchers and clinicians, the 5-year survival rate for OSCC remains unacceptably low, chiefly because of recurrences and metastases. The EMT process is a significant factor in the metastasis of OSCC, wherein OSCC cells gradually adopt the traits and activities of mesenchymal cells (323). A thorough understanding of the predisposing factors and molecular pathways associated with the initiation, development, and advancement of OSCC is crucial for developing effective strategies to battle this disease. This can be accomplished by prioritizing specific and selective therapies aimed at modulating these pathways. The main treatment targets encompass inflammatory pathways, including JAK/STAT and NF-κB, alongside cell growth and survival pathways such as RAS/RAF/MAPK, EGFR, and PI3-K/Akt/mTOR.

Another hurdle that healthcare providers and researchers face is determining the appropriate treatment modality based on the personalized individual characteristics and the molecular manifestations of the disease. Studies of oral microbiome in OSCC prove the implication of bacterial antigens as potential confounders that can interact and become involved in various developmental, apoptotic, oncogenic, mutagenesis, angiogenesis, and cell proliferation pathways (324–326). Conversely, these studies lack standardization in study design and methodology. This heterogeneity in data collection and analysis methods poses challenges for meta-analyses and the identification of reliable biomarkers. Future directions should focus on unifying and conducting comprehensive microbiome analyses to better reveal its relation and effect on OSCC. Challenges in identifying reliable epigenetic biomarkers remain an obstacle to be solved. The interaction among many pathways in OSCC is of considerable importance. This review consolidates recent literature to create a core framework for prospective clinical applications. Moreover, investigating the functions of inflammatory proteins and commensal organisms in regulating apoptosis and cancer cell growth offers a promising direction for future research. Despite numerous unresolved inquiries, our research establishes a foundation for clinical translation and implementation. We underscore the significance of cultivating strong interaction between research and clinical practice, promoting the active application of research findings in clinical environments, and advancing the evolution of personalized medicine.
